# Living different lives: Early social differentiation identified through linking mortuary and isotopic variability in Late Neolithic/ Early Chalcolithic north-central Spain

**DOI:** 10.1371/journal.pone.0177881

**Published:** 2017-09-27

**Authors:** Teresa Fernández-Crespo, Rick J. Schulting

**Affiliations:** 1 Department of Genetics, Physical Anthropology and Animal Physiology, University of the Basque Country (UPV/EHU), Leioa, Spain; 2 Research Laboratory for Archaeology and the History of Art, School of Archaeology, University of Oxford, Oxford, United Kingdom; University of Otago, NEW ZEALAND

## Abstract

Variation in burial location and treatment is often observed in the prehistoric archaeological record, but its interpretation is usually highly ambiguous. Biomolecular approaches provide the means of addressing this variability in a way not previously possible, linking the lives of individuals to their funerary treatment. Here, we undertake stable carbon and nitrogen isotope analyses on a substantial sample of 166 individuals from a series of broadly contemporary Late Neolithic/ Early Chalcolithic (3500 to 2900 cal BC) mortuary monuments (El Sotillo, Alto de la Huesera, Chabola de la Hechicera and Longar) and caves (Las Yurdinas II, Los Husos I and Peña Larga) within a very spatially restricted area of north-central Spain, with sites separated by no more than 10 km on average. This spatial and temporal proximity allows us to focus on the question at the appropriate scale of analysis, avoiding confounding variables such as environmental change, diachronic trends in the subsistence economy, etc. The results demonstrate a statistically significant difference in human δ^13^C values between those interred in caves and those placed in monuments. The difference appears to be correlated with fine-grained environmental factors (elevation/ temperature/ precipitation), suggesting that use of the landscape was being divided at a very local scale. The reasons for this partitioning may involve differential social status (e.g. those interred in caves may be of lower standing with more restricted access to the valley’s arable resources) or economic specialization (e.g. upland herding *vs*. valley farming) within the same community or, alternatively, different populations performing different funerary practices and following distinct subsistence economies in some respect. Our results contribute to a better understanding of the development of social differentiation and community specialisation on the scale of the immediate lived landscape.

## Introduction

Megalithic mortuary monuments have long dominated our perceptions of the burial practices of the Western European Neolithic. However, the last two decades have seen increasing evidence for non-monumental forms of burial across the continent, in which caves and rockshelters emerge as important alternative burial places (Britain [1–2]), Ireland [3], France [4], Portugal [5] and Spain [6–7]). The comparison is particularly germane in those regions where caves and megalithic graves coexist, raising a series of questions regarding the significance of the choice of funerary location, including potential differences in status, demographic profiles, or within/between-community identities. A problem has been that there are few opportunities to make a detailed comparison between those buried in caves and monuments within a restricted spatiotemporal setting, because the archaeological record rarely presents this ideal scenario together with large and well preserved skeletal assemblages. Such an opportunity is provided by the Rioja Alavesa region of north-central Spain in the Late Neolithic/ Early Chalcolithic (LN/EC) [8] (see S1 and S2 Appendices for a brief description of the region, period and funerary sites analyzed).

**Fig 1:**
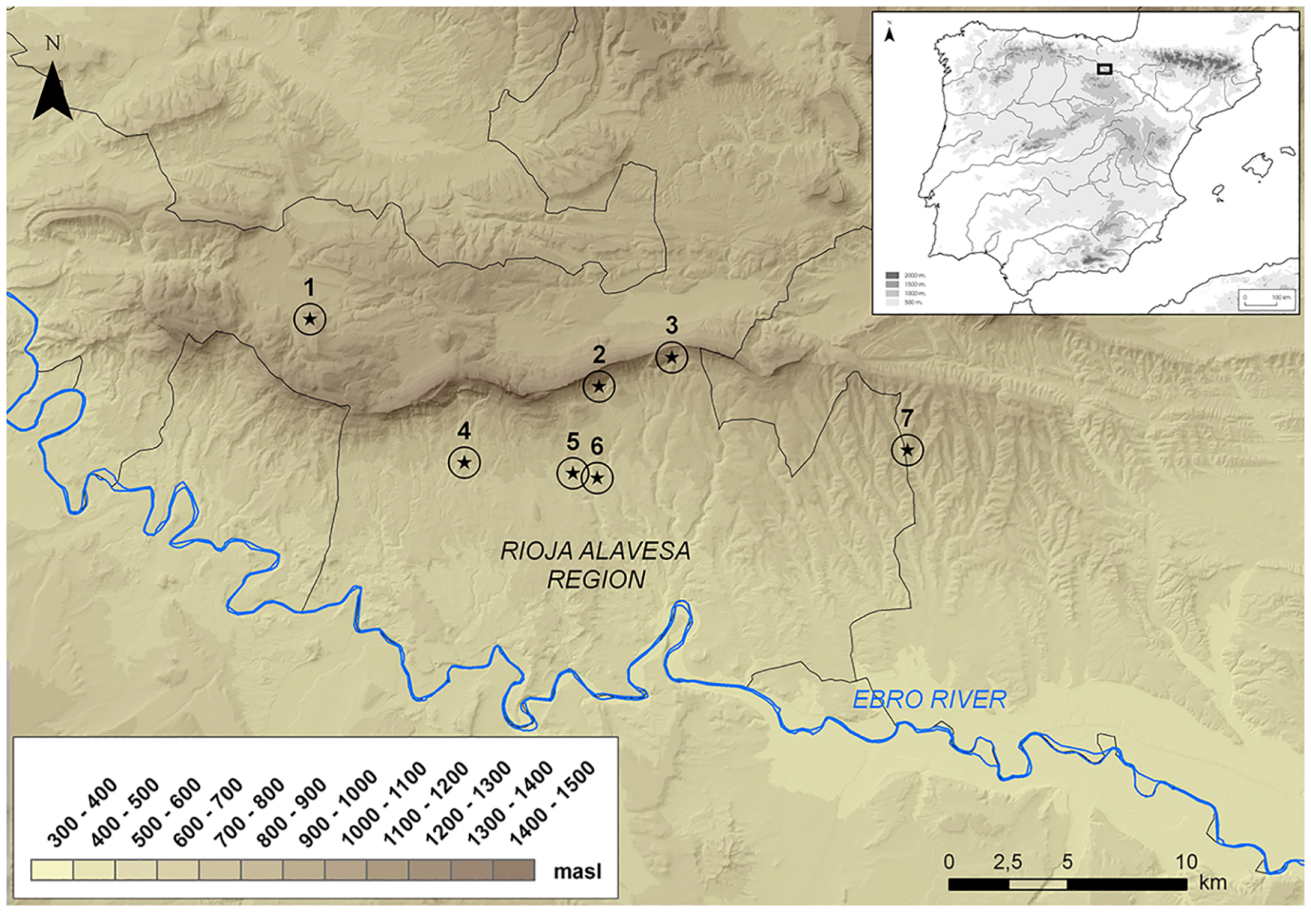
Map of the Rioja Alavesa region showing the location of the burial sites studied. *Caves and rockshelters*: 1. Las Yurdinas II, 2. Los Husos I, 3. Peña Larga; *Megalithic graves*: 4. El Sotillo, 5. Alto de la Huesera, 6. Chabola de la Hechicera, 7. Longar.

Crucially, the available radiocarbon evidence demonstrates at least partial contemporaneity in the use of the two types of burial locations in the period 3500–2900 cal BC, though the use of some dolmens extends earlier into the Middle Neolithic and later into the Late Chalcolithic and Bronze Age [9]. Regional variation is more difficult to address, as the funerary use of caves is inherently constrained by the presence of suitable landforms. Nevertheless, it is reasonably clear that the use of caves and rockshelters and of megalithic graves spatially overlaps in a number of regions, including the study area of Rioja Alavesa where both site types occur in a constrained area of some 250 km^2^ (Fig 1).

An alternative explanation could involve the use of caves and megalithic graves for different stages in a multi-stage mortuary rite spread across the landscape [10–12]. However, this does not appear to be the case in Rioja Alavesa, where good representation of all skeletal elements (even small bones of the hands and feet), the absence of signs of subaerial weathering and the frequent identification of surviving anatomical connections indicate that these assemblages represent mainly successive primary burial deposits, despite the distorted image provided by thousands of fragmented and commingled skeletal remains [13].

A final possibility is that those interred in different burial sites represent distinct social groups or populations, on whatever basis these might be defined. Discussion of this issue has recently progressed in the region, since a comparison of the demographic profiles from a number of caves and dolmens identified relatively small, but potentially meaningful differences. Infants are significantly underrepresented in megalithic graves compared to caves, and sex ratios favor women in caves and men in megalithic graves [14]. Based on these observations, the possibility that mortuary variability relates to differential status relationships within the same community does not seem unreasonable. But the alternative possibility that both groups actually belong to different communities performing different funerary practices cannot be rejected either.

In this paper, in an attempt to further investigate the meaning of different burial practices within a spatiotemporally restricted setting, we explore the use of carbon and nitrogen stable isotope analysis of human and faunal bone collagen to determine whether broad dietary differences can be detected between those interred in megalithic graves and caves. If isotopic differences can be demonstrated, it would imply that either there was a marked division (e.g. economic specialization and/or social differentiation) within a single society using both monument forms, or that two different communities, following distinct foodways, were exploiting a surprisingly restricted territory. The fact that a complementary demographic distinction has already been identified [14] could be said to favor the first interpretation.

Although δ^13^C and δ^15^N isotope analysis has been rarely implemented in Iberian prehistoric and, especially, LN/EC research until very recently [15–20], its application could provide relevant data on patterns of social differentiation, as recently observed in nearby contexts. Thus, in Late Neolithic and Copper Age central Portugal, differences in δ^15^N values between Cova da Moura and Feteira II caves and Paimogo I *tholos* were interpreted as indicating varying protein intake, which may relate to social differentiation and/or to the presence of migrants with slightly different dietary histories [21]. In the Middle Neolithic of the Languedoc region in France, individuals interred in stone-lined graves showed higher δ^15^N values than those interred in simple grave pits, suggesting a higher proportion of terrestrial meat protein consumption among the former that may imply distinct subsistence economies, i.e., greater emphasis on pastoralism in the former, and greater emphasis on arable farming in the latter [22]. Sample size in this study, however, was small, with only five stone-lined graves being compared with 32 pit graves. The implications of the present study are potentially far-reaching, since alternative burial practices are a common feature across much of prehistoric Europe, yet the underlying reasons for this variation remain poorly understood [2].

## Materials and methods

A total of 166 LN/EC human individuals was selected for analysis. They respectively derive from the caves of Las Yurdinas II (n = 49), Los Husos I (n = 9) and Peña Larga (n = 13), and the megalithic graves of El Sotillo (n = 2), Alto de la Huesera (n = 46), Chabola de la Hechicera (n = 6) and Longar (n = 41). In addition, 32 faunal samples from terrestrial herbivores and omnivores were analyzed to ascertain the variability of baseline isotopic signatures in the region. They were closely associated with the human burials and come from Los Husos I (n = 19), Peña Larga (n = 6), El Sotillo (n = 1), Alto de la Huesera (n = 2) and Chabola de la Hechicera (n = 4).

Samples were collected from the Museo de Arqueología de Álava (Bibat) in the case of Las Yurdinas II, Los Husos I, Peña Larga and Chabola de la Hechicera, from the Department of Geography, Prehistory and Archaeology of the University of the Basque Country (UPV/EHU) in the case of Alto de la Huesera and from the Museo de Navarra in the case of Longar. Sampling permissions were issued by the General Directors of Cultural Heritage of the Basque and the Navarre Governments (Spain). At present all archaeological remains are publicly stored and accessible by other researchers in the aforementioned institutions, except for those from Alto de la Huesera, which are now permanently deposited in the Museo de Arqueología de Álava (Bibat). The inventory list of the bones in their current storage locations is identical to that supplied in S1 and S2 Tables.

**Fig 2:**
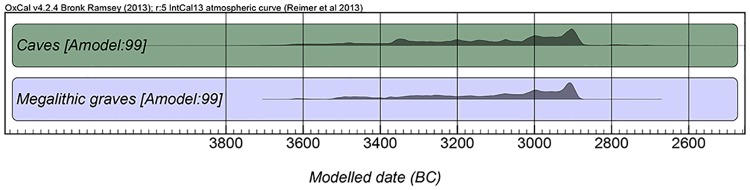
Summed probability distributions of the available LN/EC radiocarbon dates from the funerary sites under study, pooled by site type (i.e. caves and megalithic graves). The dates are modeled using OxCal 4.2.2 [24–26]. More details on radiocarbon dates are available in S1 Fig.

The funerary sites (see S2 Appendix for details) show near-contemporary use spanning approximately 600 years from ca. 3500 to ca. 2900 cal. BC (Fig 2 and S1 Fig). Given the calcareous terrain of the region, all sites had relatively well-preserved skeletal remains, even though most were found commingled, disarticulated and fragmented due to different taphonomic factors as well as to funerary practices. In the case of the human samples, mandibles were sampled, since these elements were instrumental in estimating the minimum number of individuals–thus ensuring that each sample corresponded to a unique individual–as well as providing age and sex data [13]. Furthermore, the association with dentition provides the potential for high-resolution, sequential isotopic analyses of dentine and enamel for future intra-individual dietary and mobility investigations. In a conservative approach, only those individuals older than age 7 were selected, in order to avoid complications in the interpretation of nitrogen results caused by elevated nursing signals [23] and by possible differences in early childhood diets, biasing the results towards caves, where young children occur more frequently [14]. Given the aforementioned issues of earlier use and later re-use of dolmens, only individuals that were considered contemporary to those deposited in caves and rockshelters (identified by their stratigraphic context and/or by radiocarbon dating) were sampled. In the case of the fauna, mandibles were again preferentially sampled. However, due to the scarcity of faunal remains in funerary contexts, especially in monuments, other skeletal elements were also selected, always ensuring that each sample corresponded to different individuals. Both human and animal bone samples weighed ca. 800 mg.

Collagen extraction was carried out following a modified Longin method [27] as described by Richards and Hedges [28]. Bone fragments were shot-blasted with aluminium oxide and demineralized in 0.5 M HCl solution at 5°C for one week, and then rinsed three times with deionized water until the pH became neutral. This was followed by gelatinization, being introduced to a pH3 solution over 48 h at 72°C, and later by filtering with a 5–8 mm EZEE filter. Given the good collagen preservation [29], and following standard practice at the Oxford stable isotope laboratory, the samples were not ultrafiltered. The purified solution was finally freeze-dried and lyophilized before being weighed into tin capsules and loaded into a SERCON 20/22 continuous flow ratio mass spectrometer coupled with an elemental analyzer at the Research Laboratory for Archaeology and the History of Art (RLAHA) of the University of Oxford. The carbon and nitrogen isotope ratios were measured in duplicate, and re-measured if there was a significant discrepancy between the two runs. Measurements of samples from caves and monuments were randomized, to further ensure that no minor differences in the individual runs on the mass spectrometer would affect the results. Analytical precision is ±0.2‰ (1σ) for δ^13^C and δ^15^N based on repeated analyses of internal (alanine, marine seal, cow) and international standards (IAEA 600, caffeine). Collagen preservation quality was checked according to several widely used preservation criteria: collagen yield greater than 1%, carbon content between 30% and 44% weight (wt%), nitrogen content between 11 and 16 wt%, and atomic weight C:N ratios between 2.9 and 3.6 [30–33].

Statistical analysis of results was performed using IBM SPSS software for Windows v17. Z-scores were initially calculated to detect the presence of outliers. Shapiro-Wilk tests were used to assess whether or not the data were normally distributed. For two-sample comparisons, Student’s *t*-tests were employed when the data did not depart significantly from a normal distribution; when they did, non-parametric Mann-Whitney *U*-tests were used. For more than two groups one-way ANOVA tests were conducted, since all the datasets analyzed were normally distributed with approximately equal variance. Post-hoc Tukey’s HSD tests were used to detect significant differences between sample groups. Effect size was assessed using Cohen’s *d* [34]. This statistic refers to the difference between two sample means expressed in pooled standard deviations, assuming approximately equal variances. Conventionally, a *d* value of less than 0.2 is seen as trivial, regardless of the outcome of the significance tests. Statistical power (1-*β*) was also calculated using G*Power 3.1 [35] to measure the probability that a test is correctly rejecting the null hypothesis or, in other words, that it will detect an effect when there is an effect to be detected, especially when comparing small sample sizes. Finally, Pearson’s *r*/*r*^*2*^ and Spearman’s *rho* coefficients were used to assess correlations as appropriate. A significance level of 0.05 was used for all the tests.

## Results

### Collagen preservation

The majority of the samples provided collagen yields, carbon and nitrogen percentages and C:N ratios indicating well-preserved collagen (S1 and S2 Tables). However, 13 human and faunal samples exhibited high C:N ratios (i.e. >3.6) that clearly affected the isotopic results (those from caves mainly as a result of being partially charred) and were excluded from further analyses. In addition, 67 samples exhibited collagen yields, %C and/or %N below the generally accepted limits but with C:N ratios within the accepted range [30–33]; whereas nine samples showed slightly elevated %C and/or %N, the rest of the values being acceptable. The effects of including or excluding these samples have been considered and found not to substantially alter the results, since they are consistent with other fully acceptable δ^13^C and δ^15^N measurements at each site (individual Z-scores generally < 1.0; Student’s *t*-tests, *p* > 0.05). Thus, these values do not appear to be diagenetically compromised [36] and have been retained in subsequent analyses, with the exception of one sample from Longar (LON42) with a collagen yield of 0.24%, well below the minimum threshold (0.5%) proposed by van Klinken [32]. A total of 14 samples have therefore been excluded, leaving 155 humans and 29 fauna for analysis.

### Human isotope data

The δ^13^C and δ^15^N human values cluster reasonably tightly and, as might be expected, are entirely consistent with diets focused on C_3_ plants and terrestrial animal resources, though this does still leave scope for intra- and inter-site variability (Table 1).

**Table 1:** Average δ^13^C and δ^15^N values of the human samples analyzed, by site.

Site type	Site	n	δ^13^C (‰)		δ^15^N (‰)	
			*$\bar{x}$*	*σ*	*$\bar{x}$*	*σ*
Cave	Las Yurdinas II	48	-20.1	0.3	9.2	0.5
	Los Husos I	8	-20.2	0.3	9.2	0.4
	Peña Larga	6	-20.4	0.1	9.4	0.4
	Caves combined	62	-20.2	0.3	9.2	0.5
Monument	El Sotillo	2	-20.0	<0.1	9.9	0.1
	Alto de la Huesera	46	-19.9	0.3	9.0	0.6
	Chabola de la Hechicera	6	-20.3	0.4	9.1	0.6
	Longar	39	-20.0	0.3	9.5	0.4
	Monuments combined	93	-20.0	0.3	9.3	0.6

One-way ANOVA tests indicate significant differences between sites, both in carbon (*F*_(6, 148)_ = 4.8, *p* < 0.001, *d* = 0.425, 1-*β* = 0.99) and nitrogen (*F*_(6, 148)_ = 4.7, *p* < 0.001, *d* = 0.372, 1-*β* = 0.95), with no differences in variance (Levene’s test: δ^13^C, *p* = 0.299; δ^15^N, *p* = 0.067). The application of a post hoc Tukey's HSD test (S3 Table) reveals that caves and rockshelters have fairly homogeneous isotopic values. By contrast, megalithic graves show greater inter-site heterogeneity. This is especially noteworthy in stable nitrogen isotope values, where significant differences are observed between Longar and Alto de la Huesera, whereas Chabola de la Hechicera is more similar to the caves in both δ^13^C and δ^15^N. However, given the small number of samples from this site (n = 6), little significance can be placed on this comparison, and it makes only a minimal contribution to the overall results. Grouping the samples by site type (caves n = 62 *vs*. megalithic graves n = 93) reveals clear differences in δ^13^C values with caves being slightly but statistically significantly lower (0.2‰ on average) than megalithic graves (*t* = 3.955, df = 153, *p* < 0.001, *d* = 0.649, 1-*β* = 0.98). The Cohen’s *d* value falls between what would conventionally be termed medium (ca. 0.5) and large (≥0.8) effect sizes [34], supporting the presence of a meaningful difference in average δ^13^C values. No differences are seen with regard to δ^15^N (*t* = 0.374, df = 150.7, *p* = 0.709) (S2 Fig).

#### Age-based differences

Comparisons between age groups (using age estimations made by the same researcher following the same criteria [13–14], and Buikstra and Ubelaker’s standard age categories based on skeletal maturation [37]) within each site are limited by sample size. Only at Las Yurdinas II and Longar are differences in δ^15^N values between non-adults (7–20 years) and adults (>20 years) statistically significant (*t* = 2.695, df = 46, *p* = 0.010, *d* = 0.800, 1-*β* = 0.76; and *t* = 2.076, df = 37, *p* = 0.045, *d* = 0.703, 1-*β* = 0.52, respectively) (S4 Table). If δ^15^N values from caves and megalithic graves are assessed separately, this trend is clearly detected in the former, with non-adults exhibiting slightly but significantly lower δ^15^N values compared to adults (*t* = 2.821, df = 60, *p* = 0.006, *d* = 0.745, 1-*β* = 0.80), whereas in megalithic graves it does not quite attain statistical significance in a two-tailed test, though the direction of the difference is the same (*t* = 1.798, df = 91, *p* = 0.076, *d* = 0.414, 1-*β* = 0.42). This pattern clearly emerges when combining the data from all sites (48 non-adults *vs*. 107 adults, *t* = 3.031, df = 153, *p* = 0.003, *d* = 0.523, 1-*β* = 0.85) (S3 Fig).

**Fig 3:**
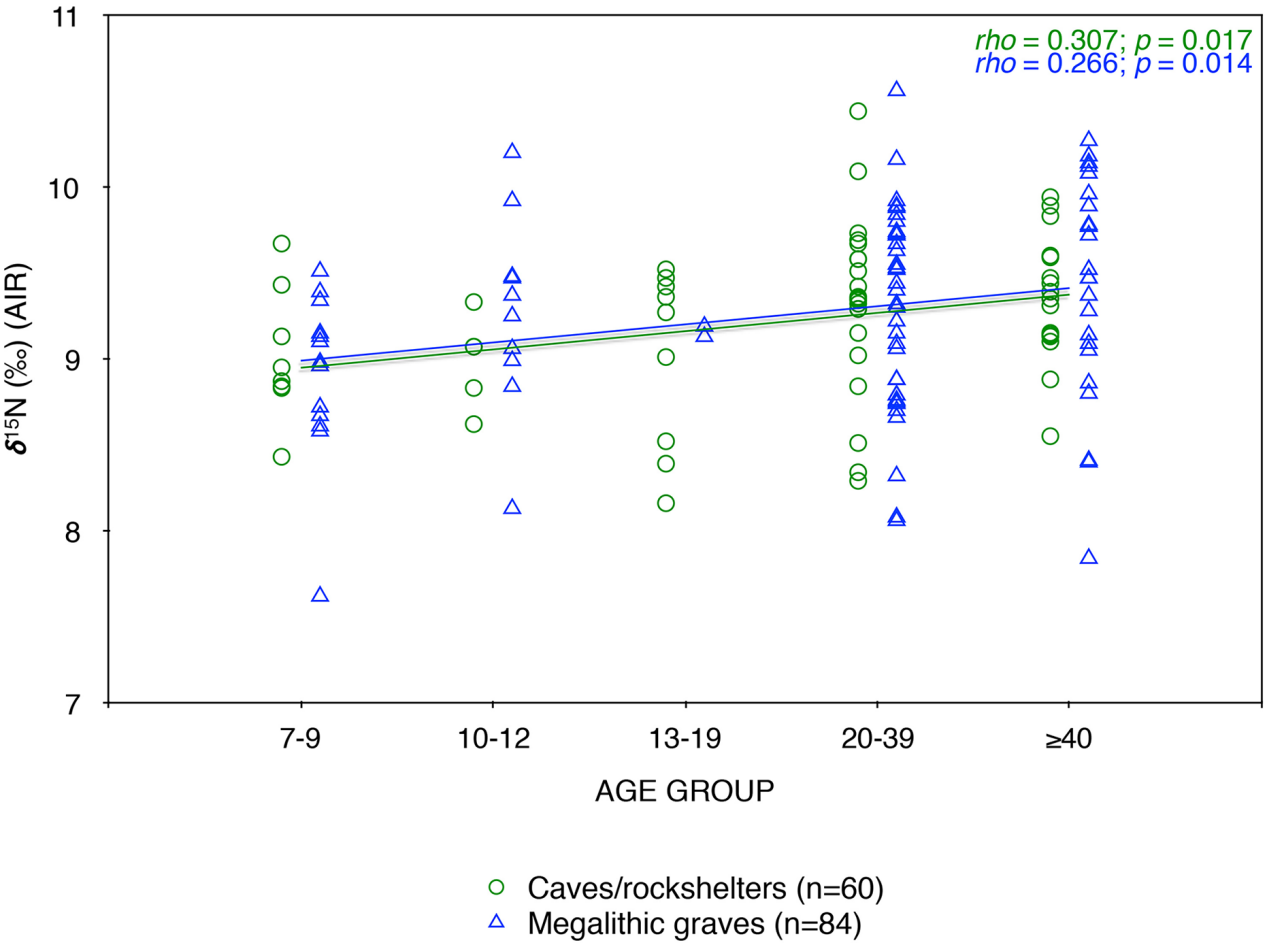
Correlation observed between human δ^15^N values and specific age groups. Values from caves and megalithic graves are distinguished. Middle and old adult age categories have been combined into ‘≥40 yrs’, since turnover rates for bone collagen become increasingly slow in older adults [40], so that stable isotope measurements will largely reflect dietary intake over the previous decades of life. For the summary statistics of the specific age groups see S5 Table.

From the perspective of social age (i.e. the culturally constructed norms of appropriate behavior and status of individuals within society for a given age [38]), it may be suggested that an earlier age, closer to 15 than 20, might be more appropriate for the transition to adulthood in archaic populations, since as a general rule the onset of fecundity in females and of sexual maturity in males define the boundary between childhood and young adult life stages [39]. However, since stable isotope signatures in bone collagen display a time lag, measurements made on a 20-year-old individual will primarily reflect the preceding years of their adolescence. Moreover, comparison of the nitrogen isotope values of juveniles and adults (>12 years) against children (7–12 years) still shows the same pattern, considering both site-types (caves: *U* = 179.5, Z = 2.404, *p* = 0.016; megalithic graves: *t* = 1.748, df = 91, *p* = 0.084) and the data from all sites (*t* = 2.508, df = 153, *p* = 0.013). Finally, using more specific age categories (7–9, 10–12, 13–19, 20–39, and ≥40) (S5 Table) clearly indicates a general progressive increase in nitrogen measurements from childhood to old age (Spearman’s *rho* = 0.291, *p* < 0.001), showing a similar correlation in caves (*rho* = 0.307, *p* = 0.017) and megalithic graves (*rho* = 0.266, *p* = 0.014) (Fig 3).

The combined δ^13^C data do not provide any clear differences between non-adults and adults, or between children *vs*. juveniles and adults. When only non-adults from each site are compared, no significant differences are seen in δ^13^C (S6 Table), but they are when the data are grouped by site type, i.e. caves *vs*. monuments (*t* = 2.117, df = 46, *p* = 0.040, *d* = 0.614, 1-*β* = 0.55). By contrast, adults do differ both between sites (one-way ANOVA, *F*_(6, 100)_ = 4.7, *p* < 0.001, *d* = 0.458, 1-*β* = 0.84)–with significant differences existing between Alto de la Huesera and Las Yurdinas II, Peña Larga and Chabola de la Hechicera, and also between Chabola de la Hechicera and Longar–, and by site type (*t* = 3.339, df = 105, *p* = 0.001, *d* = 0.665, 1-*β* = 0.91), confirming that the divergent pattern previously in δ^13^C values is driven more strongly by adults than by non-adults. As regards δ^15^N, non-adults do not show differences in variance between sites or site types, but adults do differ significantly between individual sites (*F*_(6, 100)_ = 3.6, *p* = 0.003, *d* = 0.381, 1-*β* = 0.82).

#### Sex-based differences

**Fig 4:**
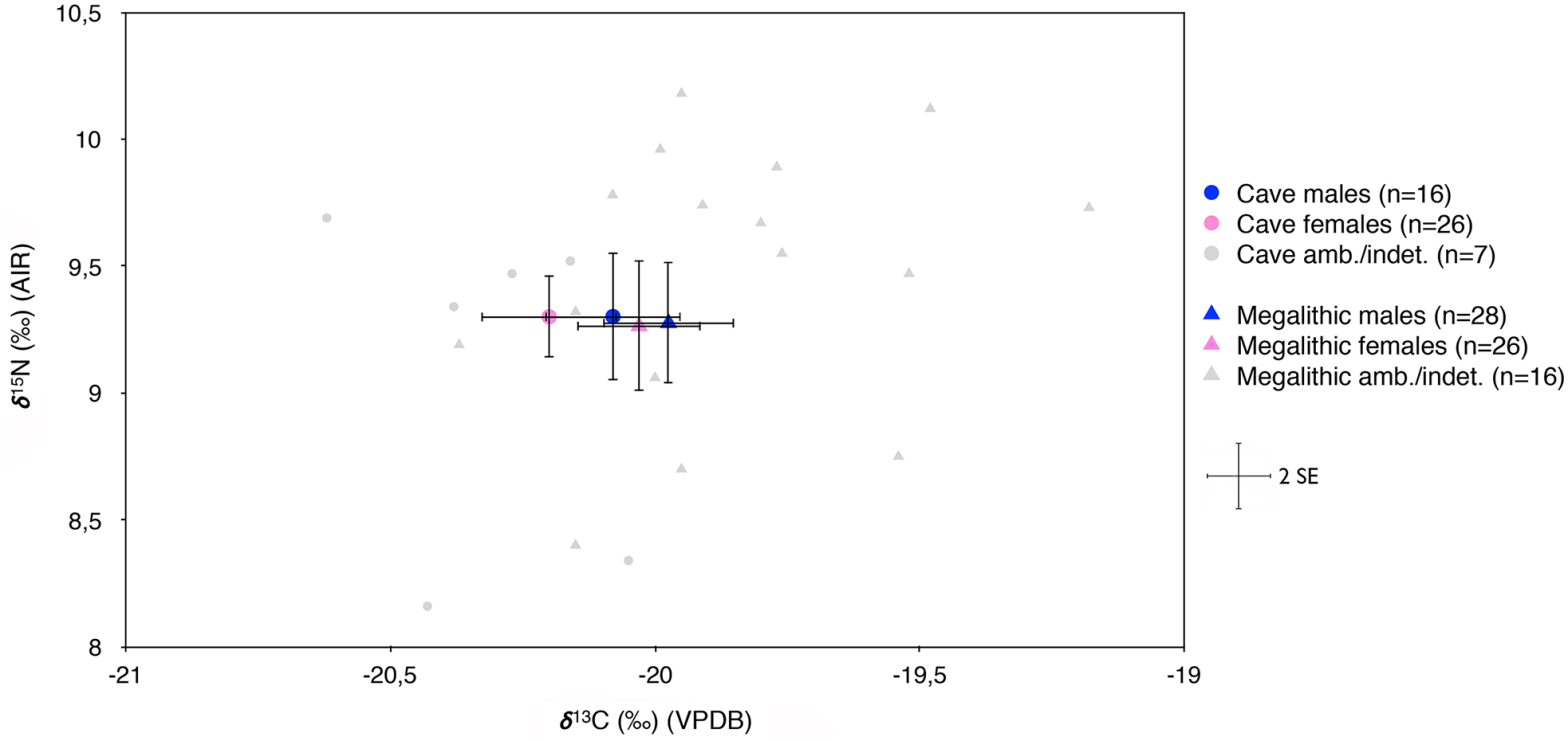
Dispersion of human δ^13^C or δ^15^N values according to sex estimation and burial type. Error bars reflect two standard errors (2SE).

Within-site analyses comparing the sexes are again limited by small sample size. With this caveat, there are no significant differences in δ^13^C or δ^15^N values between either individual sites or site types, i.e. pooling caves and megalithic graves separately (S7 Table), nor are any significant differences seen in either isotope in the combined sample (44 males *vs*. 52 females) (S4 Fig). With regard to the comparison of each sex separately by burial context, males from both burial types do not differ in either δ^13^C or δ^15^N (S8 Table). However, they do differ between sites in δ^13^C (one-way ANOVA, *F*_(4, 39)_ = 2.7, *p* = 0.047, *d* = 0.528, 1-*β* = 0.82), with post hoc tests showing significant differences between Chabola de la Hechicera and both Alto de la Huesera and Longar monuments. For women, differences between burial types approach but do not quite attain statistical significance in δ^13^C (*t* = 1.793, df = 50, *p* = 0.054, *d* = 0.551, 1-*β* = 0.50), showing higher values in megalithic graves than in caves (Fig 4); no corresponding difference is found in δ^15^N. Comparing the sites, women show significant differences in δ^15^N (*F*_(5, 46)_ = 3.0, *p* = 0.020, *d* = 0.454, 1-*β* = 0.83) but not in δ^13^C, contrary to the pattern seen in males.

### Faunal isotope data

The δ^13^C and δ^15^N ratios for herbivores and omnivores (Table 2) also are consistent with expectations for a temperate C_3_ ecosystem [41–42]. However, two red deer samples have notably higher carbon and nitrogen isotopic values than the other herbivores, which might reflect a nursing signal in one case (LHI75) and perhaps grazing in a different environment in the other (perhaps transported to the site by a hunter from further afield), since it is an adult specimen (LHI66).

**Table 2:** Average δ^13^C and δ^15^N values of the faunal samples analyzed, by species.

Site type	Species	n	δ^13^C (‰)		δ^15^N (‰)	
			*$\bar{x}$*	*σ*	*$\bar{x}$*	*σ*
Cave	*Bos taurus*	4	-21.0	0.4	4.3	1.0
	*Ovis aries / Capra hircus*	9	-20.7	0.3	4.8	1.1
	*Cervus elaphus*	4	-20.5	0.6	5.2	1.8
	*Sus domesticus*	4	-20.4	0.6	5.4	2.5
	*Sus scrofa*	1	-20.7	-	6.7	-
Monument	*Bos taurus*	3	-20.5	0.3	5.4	1.0
	*Ovis aries / Capra hircus*	3	-20.4	0.2	5.8	1.7
	*Sus domesticus*	1	-20.6	-	6.1	-

With regard to domestic herbivores (cattle and sheep/goat), higher δ^13^C and δ^15^N values are observed in specimens from megalithic graves when compared to those from caves/rockshelters but, while suggestive, the differences are not statistically significant (δ^13^C, *t* = 1.802, df = 17, *p* = 0.089, *d* = 0.854, 1-*β* = 0.37; δ^15^N, *t* = 1.741, df = 17, *p* = 0.100, *d* = 0.804, 1-*β* = 0.34) (S5 Fig). It is noteworthy, however, that the difference in δ^13^C values goes in the same direction as seen in the humans; that is, fauna from caves show lower values (Table 2). Suids exhibit wide-ranging δ^13^C and δ^15^N values, suggesting that some animals consumed a more diverse range of foods, possibly including domestic waste. The presence of a pig (LHI86) with an unusually low nitrogen value (2.6‰) is notable, as it falls outside of the range of values generally observed for omnivores (the specimen’s identification as *Sus* is secure).

## Discussion

**Fig 5:**
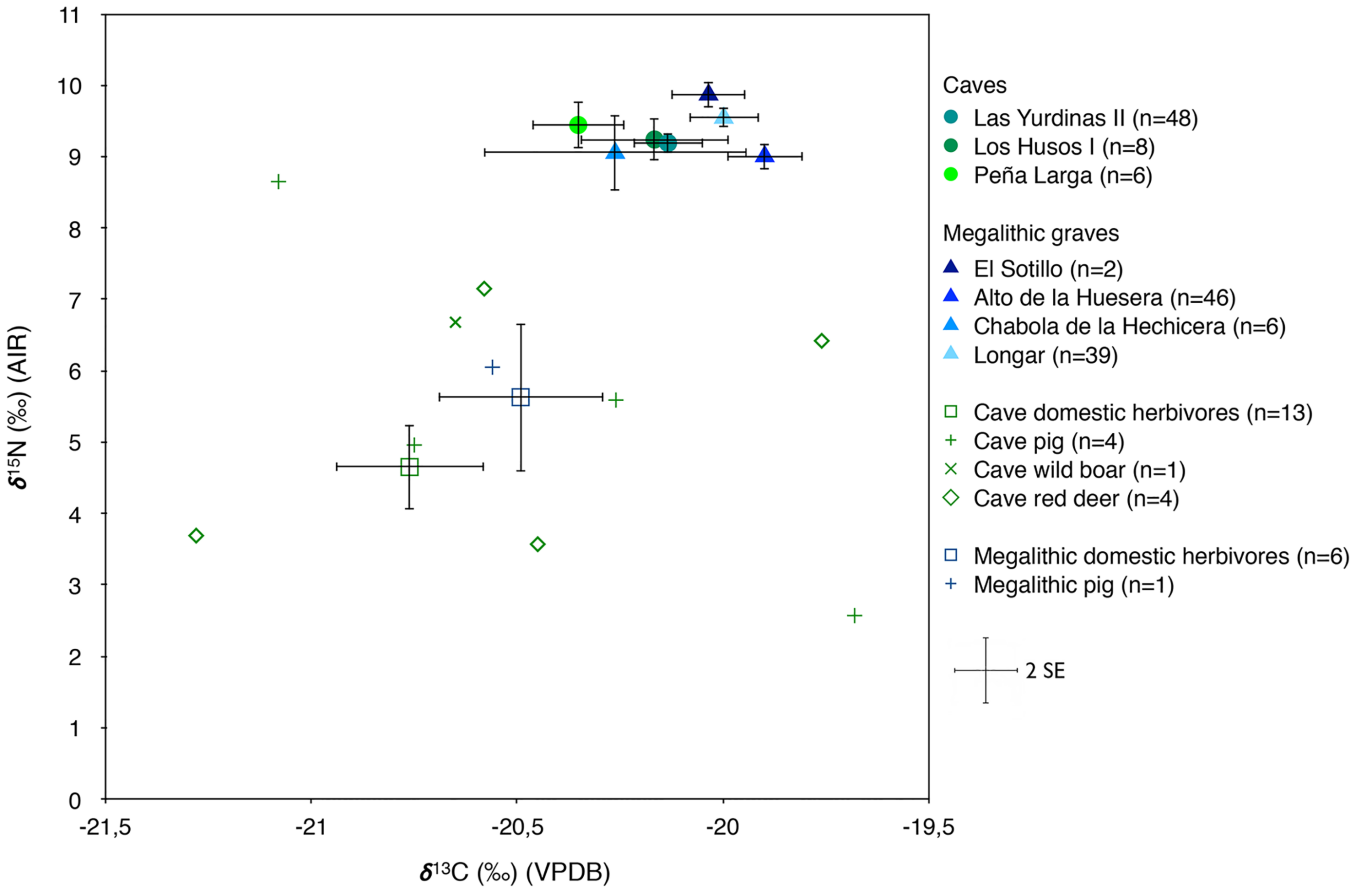
Dispersion of δ^13^C and δ^15^N isotope results obtained, grouped by site (human values) and site type (animal values). Error bars reflect two standard errors (2 SE).

The bone collagen carbon and nitrogen isotope results show an average human-faunal shift of +0.6‰ for δ^13^C and +4.5‰ for δ^15^N in caves and of +0.5‰ for δ^13^C and +3.6‰ for δ^15^N in megalithic graves. While the comparison is rather tentative due to the small size and the relatively high isotopic variability of the available faunal sample, particularly in dolmens, the values are not inconsistent with the theoretical ranges expected for a single trophic level shift [43], and suggest a C_3_ plant-based human diet with a notable contribution of terrestrial animal protein for individuals at both site types (Fig 5).

The dietary contribution of domestic plants would have come principally from wheat and barley, which are the most commonly found cereals in Iberia from the Neolithic to the Bronze Age, at which point the C_4_ plant millet first appears in the archaeological record [44]. Legumes (whose consumption would have resulted in lower δ^15^N values), berries and forest fruits and nuts (e.g. *Rosaceae*, hazelnuts) could also have featured [45–46]. Nevertheless, it is very difficult to determine the actual dietary importance of these resources as we lack the isotopic values of the relevant archaeological plants, particularly the cereals [47]. Meanwhile, animal contributions to the diet would mainly consist of meat and dairy products from cattle and sheep, though the heterogeneity in faunal age profiles suggests that they probably were not managed as specialized dairy herds [48–49]. But the dietary role of meat from domestic and wild pig and other wild animals (especially in caves, where they are more prominent (S9 Table)), which differ in being managed or hunted essentially for meat production, should not be underestimated. Finally, the results do not show any clear indications that freshwater resources contributed significantly to human diet (if so, more depleted δ^13^C and enriched δ^15^N values would be expected), though some occasional consumption cannot be rejected. Evidence in the form of barbel bones (*Barbus* sp.) and freshwater mussels (*Unio* sp.) is known from other Iberian Neolithic and Chalcolithic sites [50–51]. Given the study area’s considerable distance from the sea (>100km), the lack of any contribution of marine resources in either the isotopic or the archeozoological evidence is unsurprising. At this point it is difficult to specify the proportional contribution of the various available foods to the diet, other than to say that domestic cereals and animals appear to have dominated. In any case, the main aim of this paper is to assess the isotopic differences observed among and between site-types rather than to model precise diets.

**Fig 6:**
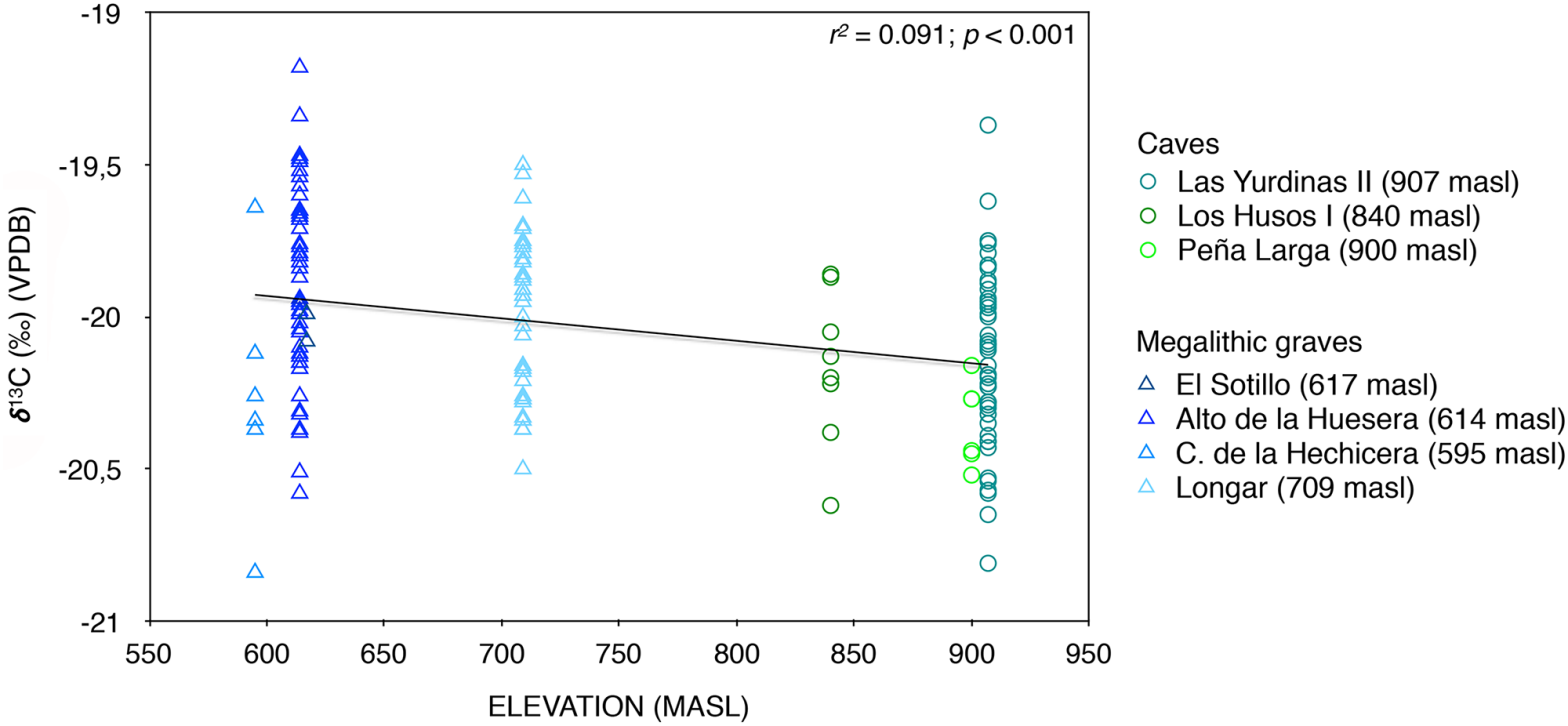
Correlation observed between human δ^13^C and δ^15^N isotope values obtained at each site and elevation (masl).

The identification of a statistically significant difference in the δ^13^C values between those interred in caves and in dolmens is an important finding of this study and so is worth considering in detail. A key question is the extent to which this relates to differences in the balance of dietary food items, or whether it is possible that there are differences in the underlying environmental isotopic baselines, even within such a small region, so that the consumption of essentially the same foods (i.e., cereals and domestic animal products) could result in slightly different stable isotope values. The lower δ^13^C values found in the humans and possibly the fauna could be consistent with the fact that the southern foothills of the Sierra de Cantabria–where caves are located–would have been more heavily forested than the valley bottom [52–53], as they are today, leading to depleted ^13^C values in plants there, and hence in animals feeding on them [54]. In addition, plant δ^13^C values in the region would have been influenced by elevation, which plays a role in conditioning temperature and precipitation [55–56] as well as forest density [57–58]. Burial caves, inherently constrained by the presence of suitable landforms, are located on the forested foothills, between ca. 800 and 900 meters above sea level (masl), with mean annual temperature (MAT) between 10.5 and 11.5°C and mean annual precipitation (MAP) between ca. 800 and 1200 mm. By contrast, dolmens are located between ca. 550 and 700 masl, with somewhat higher MAT of 12–13°C and a slightly more arid climate, with 600–700 mm MAP [59]. Each of the three ‘climate’ variables on its own is significantly correlated with the observed human δ^13^C values, though accounting for only ca. 10% of their variation. ‘Elevation’ is marginally the best predictor (*r*^*2*^ = 0.091, *p* < 0.001) (Fig 6), probably because it best encompasses the information in the other two variables, temperature (*r*^*2*^ = 0.086, *p* < 0.001) and precipitation (*r*^*2*^ = 0.050, *p* = 0.005). Although the correlations are weak (i.e. low coefficients), the observation is relevant, since it provides new evidence regarding whether or not such patterns can be detected in micro-regions like that analyzed here as well as at the much larger European scale [60–62], and can be assessed in cultural terms. In this regard, the existence of both statistically significant differences in δ^13^C values between those interred in caves and dolmens and a link between these values and climate parameters suggests a previously unsuspected complex socioeconomic differentiation among those living and burying their dead within a small region.

On the one hand, such a divergence could reflect partitioned use of a complex funerary and, by implication, living landscape by communities following distinct subsistence economies, due to the use of different catchment areas or to cultural food preferences. In this scenario, possible population movements and pressure in the Ebro valley could lie behind the use of peripheral montane areas [63], the expansion of arable fields and pastures detected through pollen analyses [53, 64] and the increase of skeletal evidence for interpersonal violence [65–68]. However, the fact that little is still known regarding the location, size and features of associated settlement sites and the current lack of studies on human mobility in the region make it difficult to assess this hypothesis.

On the other hand, it is also possible that stable isotope differences in δ^13^C results emerged within the same community, as part of an economic specialization (e.g. upland herding *vs*. valley farming) or of diverse status relationships with differential access to the most fertile lands and/or to some dietary resources. Bogaard et al. [69], for example, demonstrated on the basis of weed spectra from the Early Neolithic village of Vaihingen (Germany) that the diverse landscapes around the site reflected in differences within the community itself, and not just between settlements. Thus, it is possible that those interred in caves were of a lower social standing in some respect, with more restricted access to fertile valley plots best suited for agriculture. The hypothesis of social differentiation is not a new idea, as it has long been clear that, contrary to caves, megalithic graves only held a subset of the population [70] and their construction would involve a considerable investment of labor [71].

With regard to the observed δ^15^N variation, the results do not support climate as a driver. While not statistically significant, the higher δ^15^N values of domestic herbivores from dolmens in comparison to those from caves may be easily associated with their more common feeding (or foddering) in more intensively manured plots in the valley (indeed, an *a priori* hypothesis for higher δ^15^N values in the valley fauna could have been proposed on this basis, leading to the use of a one-tailed test which would have rejected the null hypothesis). In fact, domestic animals would have been instrumental not only as a food source but in improving fertility in garden plots–via manuring, importing nutrients (especially nitrogen) from uncultivated lands, as well as recycling crop, food and other waste–, preventing forest regeneration and, in the case of sheep, helping to reduce tillering and lodging [72]. In this respect, it is possible that the economic and symbolic role of sheep for those interred in the valley bottom’s megalithic graves is linked to the systematic use of their tibiae for the carving of bone idol-palettes, emblematic grave goods of the megalithic ritual in the mid-upper Ebro valley and across the Spanish Plateau [73–75], which have never been found in caves. Grave goods overall are very scarce in both site types, and no others show such a formal and typological complexity.

In terms of intra-group isotopic variation, a striking finding is the progressive increase in δ^15^N values from childhood to old age in both caves and megalithic graves (cf. Fig 3). This increase may be attributable to physiological processes (e.g. skeletal growth), to age related differences in diet or, more likely, to be a combination of the two [76]. However, it is also possible that lower non-adult δ^15^N values reflect compromised health status due to nutritional deficiencies or infectious diseases [21], since some skeletal evidence (e.g. non-adult cases of cribra orbitalia and rickets) may point in that direction [13]. Of course, it is also important to recognize that these children and adolescents did not reach adulthood, so that the interpretation of these results is potentially confounded by the ‘Osteological Paradox’ [77–78] (i.e., whatever differences they show may have been part of what led to their early deaths). Moreover, we also have to consider that bone collagen turnover rates are high in early infancy but decrease over the course of childhood into adolescence [79], hence reflecting different time spans—e.g., periods of stress and starvation would be expected to have much greater isotopic visibility in smaller children than in older individuals.

Moreover, the divergent pattern observed in δ^13^C remains when non-adult and adult data are analyzed separately and grouped by site type, although it is noteworthy that adults are more strongly affected. ‘Dietary’ differences (accepting that these could also refer to the consumption of food from locations with different isotopic baselines) implied in δ^13^C values may have intensified within the same community after a certain age, perhaps as a result of an individual’s acquired position. In fact, adolescents are often an important source of labor in terms of small tasks (maintaining cultivated fields, harvesting, herding, caring for siblings) and collaborating in some incipient industrial activities (pottery, mining), and their sphere of influence broadens accordingly within these archaic societies [80]. However, statistically significant differences based on caries prevalence in deciduous dentition observed between the monument of Longar and the rockshelter of San Juan ante Portam Latinam have been interpreted as a result of varying dietary practices (due to a complementary feeding belatedly introduced in the group using the dolmen after age 5–6, or, more likely, of a more sticky texture and/or higher sucrose composition for early childhood foods in the rockshelter) [46]. Thus, meaningful differences between those interred in caves and megaliths may have emerged much earlier, just after weaning, as a result of different infant-rearing practices.

There is no evidence for statistically significant differences in δ^13^C or δ^15^N between males and females within or between sites. Of course, the absence of isotopic differences between males and females cannot be interpreted as an absence of gender-based dietary distinctions, since many foods will have the same stable isotope values. However, concerning variability between site-types, while males from caves and megalithic graves show no significant differences in either carbon or nitrogen, females exhibit marginally higher δ^13^C values in megalithic graves compared to caves. While just failing to attain statistical significance, this pattern is nevertheless worth considering further.

A first possibility is that this pattern might be related to differential status relationships within the same population. Thus it may be the case that men and women from megaliths, if they were higher status graves as suspected, could both equally enjoy the valley’s food resources, whereas those interred in caves might have had less access to those presumably ‘high quality’ (socially if not nutritionally) cereals and animals from the more fertile valley-bottom fields. In this respect, statistically significant differences found in the location and incidence of oral pathologies from Longar and San Juan ante Portam Latinam were interpreted as a result of privileged access to domesticates (especially cereals) by those interred at the monument, whereas those inhumed at the rockshelter may have made greater use of wild resources (berries, forest fruits and nuts, wild animals) [46]. In this scenario, the lower δ^13^C values of women buried in caves could imply that they had more restricted access to these sources, perhaps not attaining social, economic and/or political influence as often as males [81–82], despite both presumably having lower social standing than those interred in dolmens. In this case, the hypothesis of an economic specialization (upland herding *vs*. valley farming) within the community would not make sense, unless herding was mainly a female task, as in fact has been suggested based on Neolithic rock art depictions from the lower Ebro valley and the Iberian Mediterranean coast, which show women more often engaged in the herding of domestic stock [83].

An alternative explanation, based on the idea of two different populations exploiting slightly different parts of what was essentially the same landscape, is that women’s activities would have usually been ‘hearth-centered’ and more confined to the area in and around settlements (e.g. for child care, food preparation, harvesting, manufacture of clothes, vessels and flint and bone tools) [84], so that they essentially consumed more local food. In this scenario, the δ^13^C average of the males buried in caves, intermediate between those of the women in caves and all those interred in megaliths, may suggest that they had higher mobility, probably due to herding and/or hunting activities, and so had access to a greater variety of resources from both montane and valley ecosystems.

Additional research will be required to solve these and other questions emerging from this study, including high-resolution sequential sampling of tooth dentine to determine the age at which the observed isotopic differences in bone collagen first appear and strontium isotope analysis to investigate different patterns of mobility and landscape-use in the study area. But, whatever final interpretation is supported, the stable carbon and nitrogen isotope results on human remains from Rioja Alavesa’s Late Neolithic/ Early Chalcolithic provide the first clear evidence for a division in the lifeways of those burying their dead in funerary monuments versus natural caves. The finding is exceptional because, while detecting isotopic differences between individuals within a given population (either by sex, age, grave goods, etc.) is not uncommon in the available literature [19, 85–86], what is far less frequent is to find instances where differential mortuary practices and more specifically burial site-types show a relationship to diet (or, at least, to isotopic values), especially in prehistory. And when they do, it affects δ^15^N values [21–22], as previously mentioned. Since measurements on human bone collagen reflect averaged dietary intake over a decade or more in adults, the implication is that the majority of those whose diets and/or use of the landscape differed in life were destined for burial in one or the other funerary location for many years. In fact, since δ^13^C values differed significantly in non-adults (7–20 yrs) as well as in adults, this pattern may have been in place from birth. As well as providing new insights into the lives of those using coeval funerary monuments within a small region, the results demonstrate that, at least in this area and at this time, the treatment of the dead does reflect to some extent who they were in life.

## Conclusions

Understanding variability in burial treatment is one of the key aims of funerary archaeology. The results of the present study provide another example of the new insights that can be provided by biomolecular approaches, bringing new evidence to bear on old questions. Perhaps most striking is that, even within a purely terrestrial C_3_ ecosystem, with no clear significant input from any aquatic foods, it has been possible to identify meaningful patterns in the diets of the living that determined to some extent their treatment at death. Stable carbon and nitrogen isotopic data from human and faunal remains from coeval funerary sites in the Rioja Alavesa demonstrate the existence of subtle but nevertheless significant isotopic differences among and between communities living in close proximity and burying their dead in caves and megalithic graves. These findings offer a valuable contribution to the debate on the underlying rationale behind mortuary variability in south-western Europe and suggest differences in the access to dietary resources and/or landscape use at a constrained geographic and chronological scale, with implications for the emergence of socioeconomic inequalities.

## Supporting information

S1 Appendix The Rioja Alavesa region and the Late Neolithic/ Early Chalcolithic. (DOCX)

S2 Appendix The Late Neolithic/ Early Chalcolithic funerary record in Rioja Alavesa. (DOCX)

S1 Fig Available LN/EC radiocarbon dates from the funerary sites under study (Armendáriz and Irigaray 2007; Fernández-Eraso 2007/2008; Fernández-Eraso and Mujika 2013). The dates are grouped by burial type and Bayesian modeled as phases using OxCal 4.2.2. (Bronk Ramsey 2013; Reimer et al. 2013). Los Husos I only date is treated as charcoal outlier (i.e., potentially residual, and/or having an in-built age of unknown duration) (Bronk Ramsey 2009).
(TIF)

S2 Fig Boxplot of δ^13^C and δ^15^N values for cave and megalithic humans. (TIF)

S3 Fig Boxplot of δ^13^C and δ^15^N values for human non-adults and adults. (TIF)

S4 Fig Boxplot of δ^13^C and δ^15^N values for men and women. (TIF)

S5 Fig Boxplot of δ^13^C and δ^15^N values for cave and megalithic domestic herbivores. (TIF)

S1 Table Human isotope values and bone collagen quality indicators of the samples analyzed. (DOCX)

S2 Table Faunal isotope values and bone collagen quality indicators of the samples analyzed. (DOCX)

S3 Table Post hoc Tukey's HSD tests used to assess heterogeneity in δ^13^C and δ^15^N values between sites. (DOCX)

S4 Table Summary statistics of human isotopic values grouped in non-adult (7–20 years) and adult (>20 years) age categories and statistical results obtained from comparing the mean values of both groups among sites, by site-type and in total. (DOCX)

S5 Table Summary statistics of human isotopic values grouped in specific age categories (7–9, 10–12, 13–19, 20–39, ≥40). (DOCX)

S6 Table Statistical results obtained from comparing the mean values between non-adults (7–20 years) and between adults (>20 years) separately between sites and by site-type (see S4 Table for the summary statistics of the groups being compared). (DOCX)

S7 Table Summary statistics of human isotopic values grouped according to sex estimation and statistical results obtained from comparing the mean values of males and females among sites, by site-type and in total. (DOCX)

S8 Table Statistical results obtained from comparing the mean values between males and between females separately between sites and by site-type (see S7 Table for the summary statistics of the groups being compared). (DOCX)

S9 Table Minimum number of individuals (MNI) of the ungulate remains recovered from the sites analyzed. (DOCX)
