# Supplementary material for: Living different lives: Early social differentiation identified through linking mortuary and isotopic variability in Late Neolithic/ Early Chalcolithic north-central Spain
Source: PLoS One. 2017 Sep 27;12(9):e0177881. doi: 10.1371/journal.pone.0177881 (PMC5643145; doi:10.1371/journal.pone.0177881)
Supplement: S1 Appendix — (DOCX) [file pone.0177881.s001.docx]

**S1 Appendix from Fernández-Crespo and Schulting, “Living different lives: early social differentiation identified through linking mortuary and isotopic variability in Late Neolithic/ Early Chalcolithic north-central Spain”**

**The Rioja Alavesa region and the Late Neolithic/Early Chalcolithic**

Rioja Alavesa is a confined region located in the mid-upper Ebro Valley (north-central Spain), belonging to Álava province (Basque Country). It is delimited by the steep Cretaceous limestone range of the Sierra de Cantabria-Toloño (900-1450 masl) to the north and by the middle course of the Ebro river to the south (Fig 1). The landscape is predominantly one of gentle south-facing slopes, mainly composed of Tertiary sandstone deposits, occasionally covered with thin Quaternary deposits [1]. The region is characterized by a Continental Mediterranean climate, the range itself acting as a barrier against Atlantic influences from the northern part of Álava and thus making the existence of different ecosystems possible both latitudinally and altitudinally [2].

The period spanning the mid-4th to early-3rd millennia cal. BC – culturally coincident with the LN/EC – experienced a temperate, relatively arid climate, dominated by meso-thermophilic mixed forests, principally composed by hazel, birch, alder, lime, ash and willow [3]. Also present are oak forests, together with pine, yew, bush, shrubs like heather and juniper and arid grasses (Artemisia, Centaurea). A decrease in the percentage of tree pollen and the appearance of ruderal and nitrophilic species (Chenopodiaceae, *Plantago* sp., *Urticadioica*) have been interpreted as a progressive anthropogenic depletion of forests and the expansion of arable fields and pastures [4-5]. Moreover, domestic cereal pollen appears in the local botanical record (although in low percentages) and there is micromorphological evidence of ovicaprid stalling in some rockshelters of the range [6-7].

Perhaps promoted by favorable climatic conditions, the period being considered here sees both a proliferation of open-air settlements, especially in the lowlands where the terrain is more suitable for agriculture [8], and a diversification of burial sites [9]. Thus, while passage tombs built in the Middle Neolithic continue to be in use and *ex-novo* monuments continue to be constructed in the valley, caves and rockshelters on both sides of the Sierra Cantabria-Toloño also start being used as funerary spaces [10]. The occupation and/or more extensive use of peripheral montane areas where the caves are located could suggest population growth spilling out of the main river valley. A pastoral economy would present an appropriate use of this landscape [11]. However, presuming an exclusive link between pastoral specialization and people using caves as burial places just on the basis of their chronological coincidence is rather speculative [12].

There is accumulating evidence that the LN/EC witnessed an increase in social unrest that may relate to demographic pressure and/or to incipient social complexity [13]. Evidence of interpersonal violence takes the form of arrowhead injuries in human skeletal remains and, less frequently, parry fractures and depressed cranial fractures [14-17]. Moreover, the first appearance of metal objects and some prestige items (votive polished stone axes, carved bone idol-palettes, bone and stone beads, etc.) in funerary contexts could be linked to the development of hierarchical societies and the appearance of social inequality in the region [18]. Thus, it is possible that all these social changes are associated with greater heterogeneity in access to subsistence resources.

**References**

1. Arnedo F, Urbina A. La Rioja. Espacio y Sociedad. Geografía, vol. 1. Logroño: Fundación Caja Rioja; 2000.
2. Núñez E, Martínez J. El clima de La Rioja. Análisis de precipitaciones y temperaturas. Logroño: Gobierno de La Rioja; 1991.
3. Rofes J, Zuluaga MC, Murelaga X, Fernández-Eraso J, Bailon S, Iriarte MJ, et al. Paleoenvironmental reconstruction of the early Neolithic to middle Bronze Age Peña Larga rock shelter (Álava, Spain) from the small mammal record. Quat Res. 2013; 79: 158-167.
4. Pérez-Díaz S, Ruiz M, López JA, Zapata L. Dinámica vegetal y antropización en la Sierra de Cantabria (Álava) desde el Neolítico a la Edad del Bronce. Polen. 2010; 20: 25-40. DOI: 10.14201/pol.v20i0.8916.
5. Pérez-Díaz S, López-Sáez JA, Galop D. Vegetation dynamics and human activity in the Western Pyrenean Region during the Holocene. Quat Int. 2015; 364: 65-77.
6. Polo A, Fernández-Eraso J. Aportación de la micromorfología a la determinación de los rediles prehistóricos en el alto valle del Ebro: el caso del Neolítico de Los Husos II (Elvillar, Álava). Rev. C.&G. 2008; 22(3-4): 159-171.
7. Iriarte MJ. Vegetation landscape and the anthropization of the environment in the central sector of the Northern Iberian Peninsula: Current status. Quat Int. 2009; 200: 66-76.
8. Andrés MT. Fases de implantación y uso dolménico en la Cuenca Alta y Media del Ebro. In: Rodríguez Casal A, editor. O Neolítico Atlántico e as orixes do Megalitismo (Santiago de Compostela, 1996). Santiago de Compostela: Universidad de Santiago de Compostela; 1997. pp. 431-444.
9. Fernández-Eraso J, Mujika J, Zapata L, Iriarte MJ, Polo-Díaz A, Castaños P, et al. Beginnings, settlement and consolidation of the production economy in the Basque region. Quat Int. 2015; 364: 162-171.
10. Fernández-Eraso J, Mujika JA. La estación megalítica de la Rioja Alavesa: cronología, orígenes y ciclos de utilización. Zephyrus. 2013; LXXI: 89-106.
11. Andrés MT. Comportamiento funerario en el Neolítico y Eneolítico: sociedad e ideología. In: Santos J, editor. Los tiempos antiguos en los territorios pirenaicos. Anejos de Veleia, Series acta 8. Vitoria-Gasteiz: Universidad del País Vasco (UPV/EHU); 2009. pp. 11-36
12. Andrés MT. Concepto y análisis del cambio cultural: su percepción en la materia funeraria del Neolítico y Eneolítico. Monografías Arqueológicas 42. Zaragoza: Departamento de Ciencias de la Antigüedad, Universidad de Zaragoza; 2005.
13. Delibes G. Ritos funerarios, demografía y estructura social entre las comunidades neolíticas de la submeseta norte. In: Fábregas R, Párez F, Fernández C, editors. Arqueoloxía da Morte na Península Iberica desde as Orixes ata o Medievo. Xinzo de Limia: Concello de Xinzo de Limia; 1995. pp. 61-94.
14. Armendáriz J, Irigaray S, Etxeberria F. New evidence of prehistoric arrow wounds in the Iberian Peninsula. Int J Osteoarchaeol. 1994; 4: 215-222. DOI: 10.1002/oa.1390040306
15. Vegas JI, Armendáriz A, Etxeberria F, Fernánez MS, Herrasti L. Prehistoric violence in northern Spain: San Juan ante Portam Latinam. In: Schulting RJ, Fibiger L, editors. Sticks, Stone and Broken Bones: Skeletal Evidence for Interpersonal Violence in Neolithic Europe. Oxford University Press: Oxford; 2012. pp. 265-302.
16. Fernández-Crespo T. An arrowhead injury in a Late Neolithic/Early Chalcolithic human cuneiform from the rockshelter of La Peña de Marañón (Navarre, Spain). Int J Osteoarchaeol. 2016; 26: 1024-1033. DOI: 10.1002/oa.2513
17. Fernández-Crespo T. New Evidence of Early Chalcolithic Interpersonal Violence in the Middle Ebro Valley (Spain): Two Arrowhead Injuries from the Swallet of Las Yurdinas II. Int J Osteoarchaeol. 2017; 27: 76-85. DOI: 10.1002/oa.2445
18. Guerra E, Delibes G, Zapatero P, Villalobos R. *Primus inter pares*: estrategias de diferenciación social en los sepulcros megalíticos de la Submeseta Norte española. BSAA Arqueología. 2009; LXXV: 41-65.
