# Supplementary material for: Living different lives: Early social differentiation identified through linking mortuary and isotopic variability in Late Neolithic/ Early Chalcolithic north-central Spain
Source: PLoS One. 2017 Sep 27;12(9):e0177881. doi: 10.1371/journal.pone.0177881 (PMC5643145; doi:10.1371/journal.pone.0177881)
Supplement: S2 Appendix — (DOCX) [file pone.0177881.s002.docx]

**S2 Appendix from Fernández-Crespo and Schulting, “Living different lives: early social differentiation identified through linking mortuary and isotopic variability in Late Neolithic/ Early Chalcolithic north-central Spain”**

**The Late Neolithic/Early Chalcolithic funerary record in Rioja Alavesa**

The LN/EC funerary record of Rioja Alavesa consists of four funerary caves or rockshelters (San Juan ante Portam Latinam, Los Husos I and II, and Peña Larga) and eight megalithic graves (El Montecillo, Layaza, El Sotillo, San Martín, Alto de la Huesera, Chabola de la Hechicera, El Encinal and Los Llanos). The group is completed by two caves (Las Yurdinas II and La Peña de Marañón) and two monuments (La Cascaja and Longar) that are located just outside the limits of the region but still form part of the same natural and cultural environment. Of these sites, three dolmens (El Montecillo, Layaza and El Encinal) were found looted and have very limited human remains; two caves (Los Husos II and La Peña de Marañón) are almost entirely represented by cremated remains and so are not suitable for collagen-based isotopic analysis; and four sites are still undergoing anthropological (Los Llanos) or isotopic analysis (La Cascaja, San Martín and San Juan ante Portam Latinam). The study presented here, therefore, focuses on human and animal remains from three caves (Las Yurdinas II, Los Husos I and Peña Larga) and four megalithic graves (El Sotillo, Alto de la Huesera, Chabola de la Hechicera and Longar) whose contemporary use spans approximately 600 years from ca. 3500 to ca. 2900 cal. BC (Fig 2 and S1 Fig).

Las Yurdinas II (Peñacerrada, Álava). The site is a small cave located in the northern slope of Sierra de Cantabria-Toloño mountain range (907 masl). Only one archaeological layer was found, containing a minimum of 90 individuals (37 non-adults and 53 adults). Grave goods were scarce, consisting of several arrowheads and a few shell pendants and necklace beads. There were no associated contemporary faunal remains. Both the grave goods and the available radiocarbon dates on human bone are consistent with a LN/EC chronology, ca. 3350-2900 cal. BC [1].

Los Husos I (Elvillar, Álava). This large rockshelter is located in the southern slope of Sierra de Cantabria-Toloño (840 masl). Excavations revealed a stratigraphy consisting of several layers spanning the Neolithic to Late Roman periods [2-3]. Nevertheless, the funerary use of the site was restricted to layer III, which contained at least 18 individuals (11 non-adults and seven adults) together with some stone tools (end-scrapers, scrapers, sickle blades, polished axes), pottery, a red deer antler pick, a bone palette, a necklace bead and diverse faunal remains. The site has only a single radiocarbon date on charcoal with a large error term, but both the sequence and the grave goods clearly point to a LN/EC chronology.

Peña Larga rockshelter (Cripán, Álava). The site is also situated to the south of the range (900 masl). It contains several archaeological layers from the Early Neolithic to the Bronze Age, but the only funerary deposit corresponds to layer III, dated to the LN/EC ca. 3400-3000 cal. BC (4-5). The remains of a minimum of 31 individuals (12 non-adults and 19 adults) were recovered, as well as grave goods consisting of pottery, three stemmed and barbed arrowheads, some bone punches and diverse faunal remains.

El Sotillo (Laguardia, Álava). It is a passage tomb located in the Ebro river valley (617 masl). It contained at least 17 individuals (two non-adults and 15 adults) together with several flint arrowheads and blades, a polished stone axe, a bone punch, a copper arrowhead, diverse pottery sherds and very limited faunal remains [6]. In view of the absence of stratigraphic data, radiocarbon dates were obtained, proving that only two individuals directly dated to the LN/EC, ca. 3100-2900 cal. BC [7]. These are included in the present study.

Alto de la Huesera (Laguardia, Álava). This passage tomb is also situated in the Ebro valley (614 masl), with a chamber exhibiting two chronologically distinct burial layers, in this case divided by a stone paving. Both the inferior layer [7] and the corridor (J. Fernández-Eraso and J. Mujika, pers. comm.) show that at least 56 individuals (18 non-adults and 38 adults) were interred during the LN/EC, dated ca. 3350-2900 cal. BC on human bone. The few associated finds consists of some flint tools, a few pottery sherds, diverse necklace beads and scarce faunal remains [8]. The superior layer dates to the Late Chalcolithic, ca. 2800-2300 cal. BC, and as such does not feature in the present study.

Chabola de la Hechicera (Elvillar, Álava). This is a passage tomb located close to the previous site (595 masl), and contained a minimum of 39 individuals (nine non-adults and 30 adults). Grave goods comprised diverse flint arrowheads, end-scrapers and blades, some pottery sherds, one carved bone idol-palette, several necklace beads and very few faunal remains [9]. As in the case of El Sotillo, the lack of clear stratigraphic data prevents from delimiting different phases of use. Nevertheless, recent radiocarbon dates determined that at least seven individuals belonged to the LN/EC, ca. 3650-2900 cal. BC [7], of which six are included in the present study.

Longar (Viana, Navarre). The monument is situated in the Ebro valley but at a higher altitude (709 masl), and has an unusual structure for the region, consisting of a horseshoe shape burial chamber built of mortarless ashlar and covered by two huge slabs, an access door cut in a vertical slab and a megalithic corridor [10]. It holds a minimum of 114 individuals. The finds were mainly limited to flint arrowheads, four of which were found embedded in human bone. Available radiocarbon dates of ca. 3500-3000 cal. BC on human bone are consistent with the LN/EC.

**References**

1. Fernández-Eraso J, editor. Las Yurdinas II: Un depósito entre finales del IV y comienzos del III milenio BC. Memorias de Yacimientos Alaveses 8. Vitoria-Gasteiz: Diputación Foral de Álava; 2003.
2. Apéllaniz JM. El Grupo de Los Husos durante la prehistoria con cerámica del País Vasco. Estudios de Arqueología Alavesa. 1974; 7: 1-409.
3. Fernández-Eraso J. La secuencia del Neolítico en la Rioja Alavesa desde su origen hasta las primeras edades del metal. In: Fernández-Eraso J, Santos J, Barandiarán I, editors. Homenaje a Ignacio Barandiarán Maestu. Veleia 24-25.Vitoria-Gasteiz: Universidad del País Vasco (UPV/EHU); 2007/2008. pp. 669-687.
4. Fernández-Eraso J, editor. Peña Larga. Memorias de Yacimientos Alaveses 4. Vitoria-Gasteiz: Diputación Foral de Álava; 1997.
5. Rofes J, Zuluaga MC, Murelaga X, Fernández-Eraso J, Bailon S, Iriarte MJ, et al. Paleoenvironmental reconstruction of the early Neolithic to middle Bronze Age Peña Larga rock shelter (Álava, Spain) from the small mammal record. Quat Res. 2013; 79: 158-167.
6. Barandiarán JM, Fernández Medrano D. Excavaciones en el domen del Sotillo (Laguardia, Álava). Boletín de la Institución Sancho el Sabio. 1964; VIII (1-2): 28-41.
7. Fernández-Eraso J, Mujika JA. La estación megalítica de la Rioja Alavesa: cronología, orígenes y ciclos de utilización. Zephyrus. 2013; LXXI: 89-106.
8. Fernández-Eraso J, Mujika JA. Dólmenes de La Rioja Alavesa: El Montecillo (Villanueva de Álava), Chabola de la Hechicera (Elvillar) y Alto de la Huesera (Laguardia). Arkeoikuska 2011; 2010: 164-173.
9. Apellániz JM, Fernández Medrano D. El sepulcro de galería segmentada de la Chabola de la Hechicera (Elvillar, Álava).Excavación y restauración. Estudios de Arqueología Alavesa. 1978; 9: 141-224.
10. Armendáriz J, Irigaray S. El sepulcro megalítico de Longar. In: Hurtado MA, Cañada F, Sesma J, García J, editors. La tierra te sea leve. Arqueología de la muerte en Navarra. Pamplona: Gobierno Foral de Navarra; 2007. pp. 73-78.
