# Supplementary figures and images for: Living different lives: Early social differentiation identified through linking mortuary and isotopic variability in Late Neolithic/ Early Chalcolithic north-central Spain
Source: PLoS One. 2017 Sep 27;12(9):e0177881. doi: 10.1371/journal.pone.0177881 (PMC5643145; doi:10.1371/journal.pone.0177881)

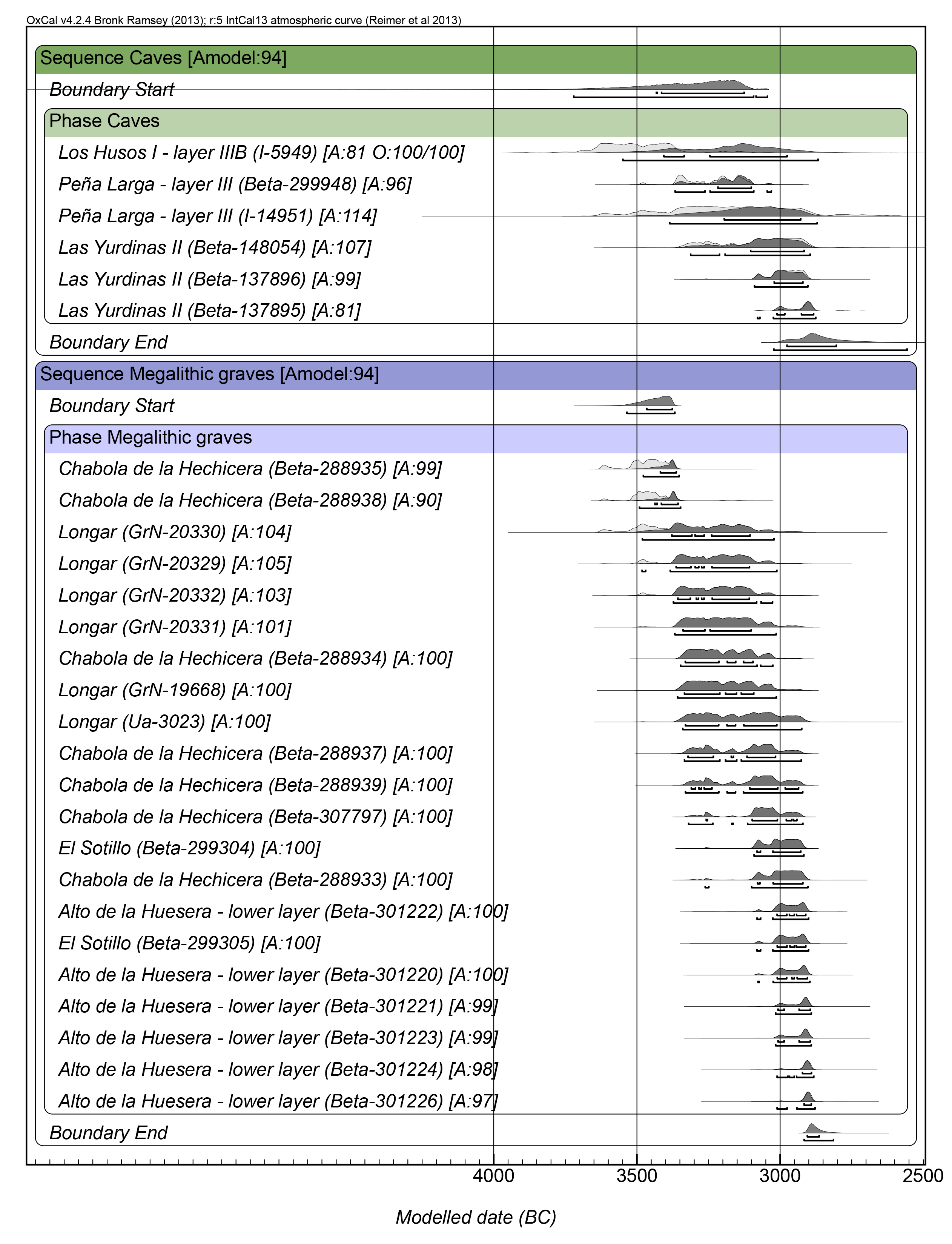

Supplement: S1 Fig — The dates are grouped by burial type and Bayesian modeled as phases using OxCal 4.2.2. (Bronk Ramsey 2013; Reimer et al. 2013). Los Husos I only date is treated as charcoal outlier (i.e., potentially residual, and/or having an in-built age of unknown duration) (Bronk Ramsey 2009). (TIF) [file pone.0177881.s003.tif]

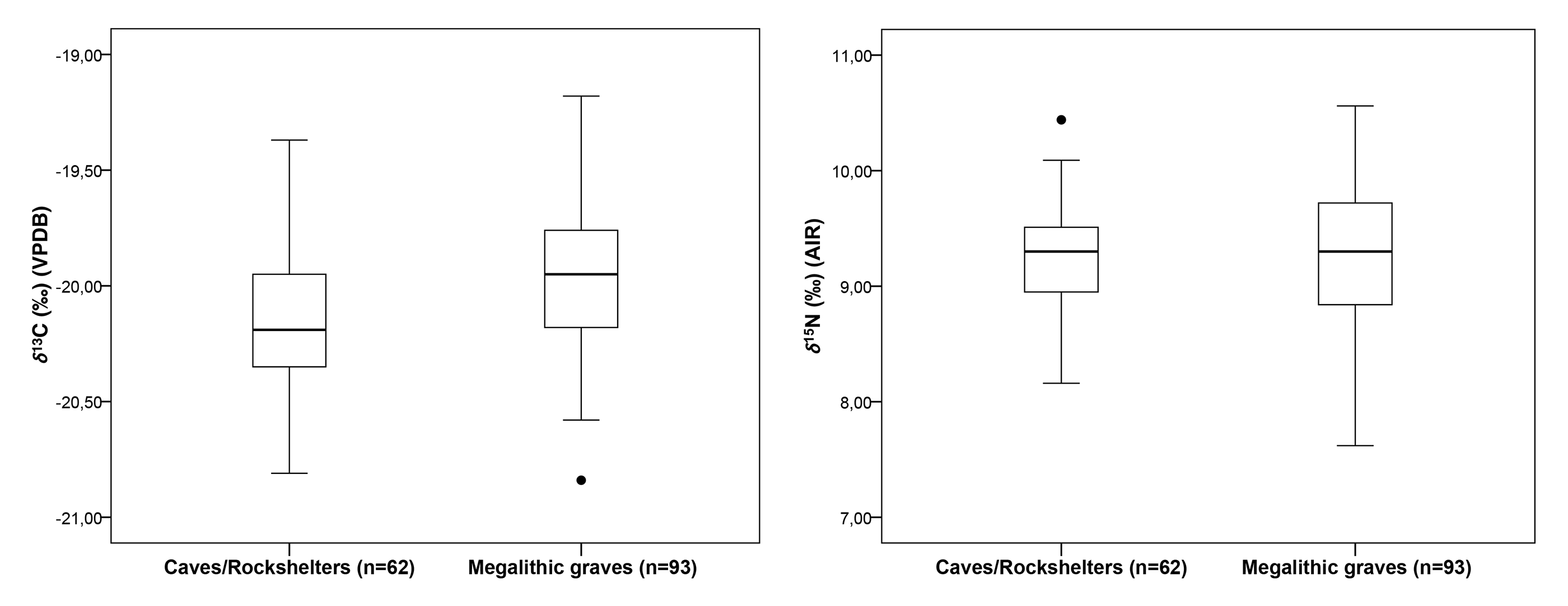

Supplement: S2 Fig — (TIF) [file pone.0177881.s004.tif]

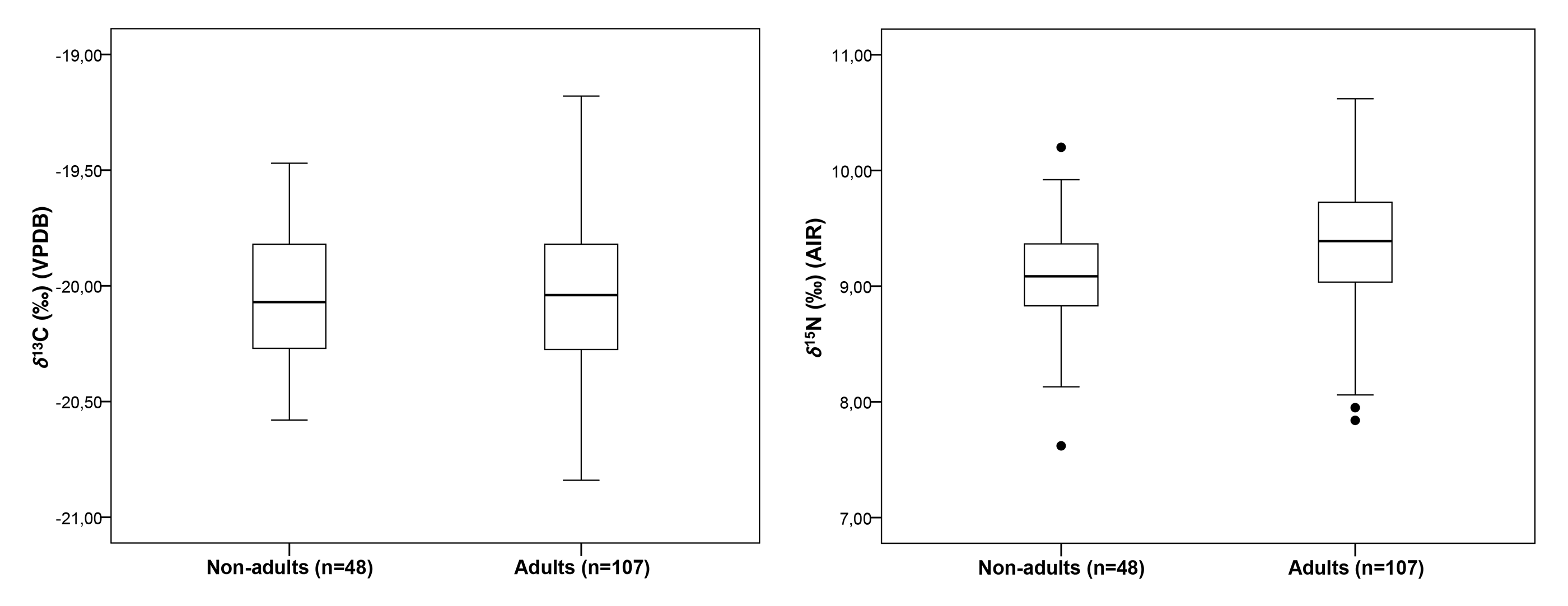

Supplement: S3 Fig — (TIF) [file pone.0177881.s005.tif]

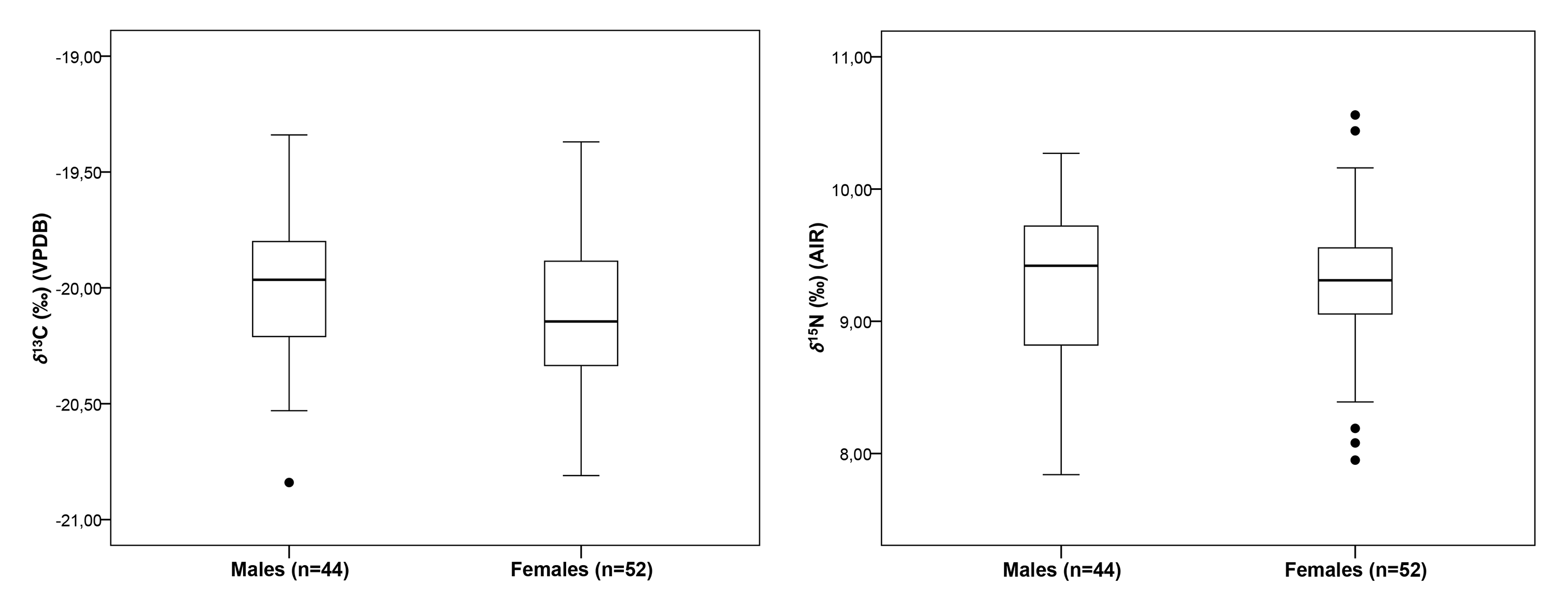

Supplement: S4 Fig — (TIF) [file pone.0177881.s006.tif]

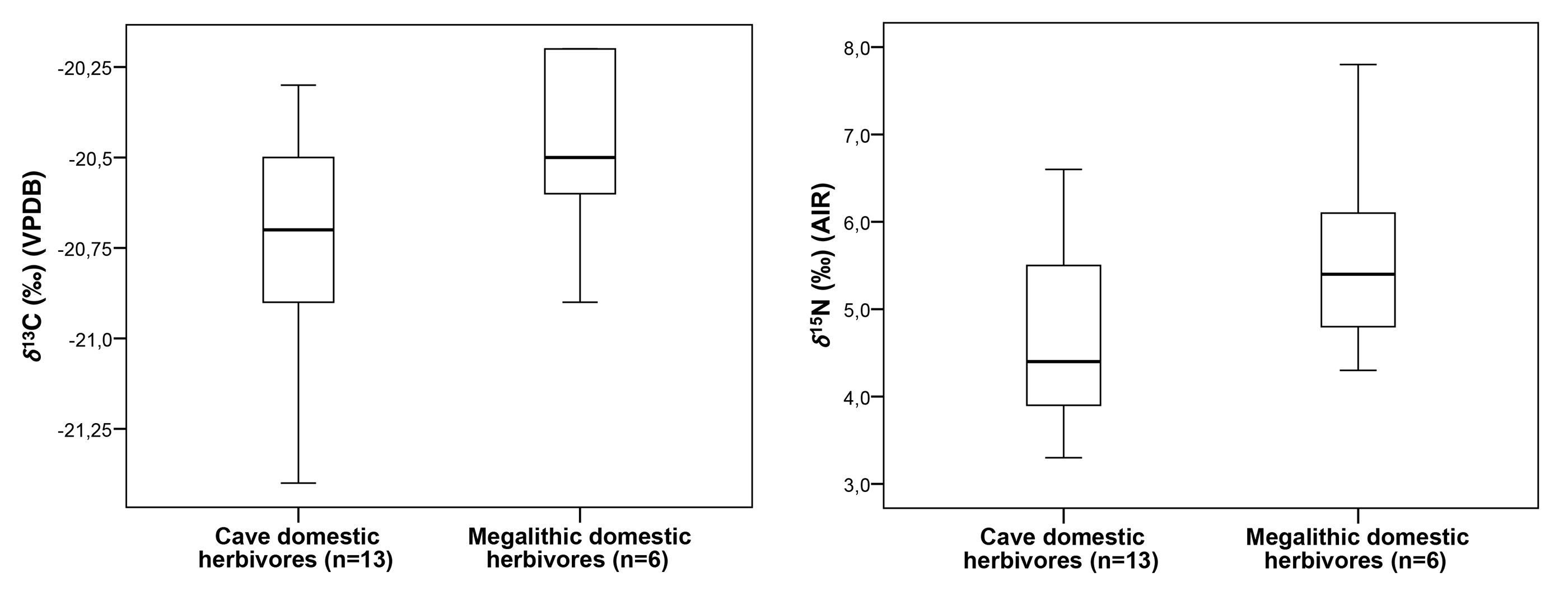

Supplement: S5 Fig — (TIF) [file pone.0177881.s007.tif]
