# Supplementary material for: Living different lives: Early social differentiation identified through linking mortuary and isotopic variability in Late Neolithic/ Early Chalcolithic north-central Spain
Source: PLoS One. 2017 Sep 27;12(9):e0177881. doi: 10.1371/journal.pone.0177881 (PMC5643145; doi:10.1371/journal.pone.0177881)
Supplement: S1 Table — (DOCX) [file pone.0177881.s008.docx]

| **S1 Table. Human isotope values and bone collagen quality indicators of the samples analyzed.** | | | | | | | | | | | | |
| --- | --- | --- | --- | --- | --- | --- | --- | --- | --- | --- | --- | --- |
| Site | Type^1^ | Sample | Inv.^2^ | Age^3^ | Sex^4^ | Element | %Col^5^ | %C^5^ | %N^5^ | C:N^5^ | δ^13^C (‰) | δ^15^N (‰) |
| Las Yurdinas II | C/RS | LYII14 | 15118 | C (7±2) | ? | Mandible | 2.0 | 34.7 | 12.4 | 3.3 | -20.2 | 8.9 |
| Las Yurdinas II | C/RS | LYII12 | 15137 | C (8±2) | ? | Mandible | 4.9 | 40.0 | 14.4 | 3.2 | -19.8 | 9.7 |
| Las Yurdinas II | C/RS | LYII13 | 15129 | C (8±2) | ? | Mandible | 6.2 | *54.7* | *20.1* | 3.2 | -20.3 | 8.8 |
| Las Yurdinas II | C/RS | LYII11 | 15083 | C (9±2) | ? | Mandible | 3.5 | *46.5* | *16.9* | 3.2 | -19.8 | 9.4 |
| Las Yurdinas II | C/RS | LYII54 | 15117 | C (9±2) | ? | Mandible | 9.0 | 37.1 | 13.3 | 3.3 | -20.1 | 8.4 |
| Las Yurdinas II | C/RS | LYII55 | 15150 | C (9±2) | ? | Mandible | 21.8 | 40.8 | 14.8 | 3.2 | -20.3 | 8.9 |
| Las Yurdinas II | C/RS | LYII52 | n/a | C (11±2) | ? | Mandible | 18.9 | *45.1* | *16.2* | 3.2 | -19.8 | 8.8 |
| Las Yurdinas II | C/RS | LYII9 | 15130 | C (12±3) | ? | Mandible | 1.4 | 36.7 | 13.0 | 3.3 | -20.3 | 8.6 |
| Las Yurdinas II | C/RS | LYII10 | 15092 | C (12±3) | ? | Mandible | 1.8 | 40.2 | 14.4 | 3.3 | -19.8 | 9.1 |
| Las Yurdinas II | C/RS | LYII22 | n/a | C (12±3) | ? | Mandible | 1.1 | 31.5 | 11.0 | 3.3 | -20.2 | 9.3 |
| Las Yurdinas II | C/RS | LYII23 | n/a | C (12±3) | ? | Mandible | 1.3 | 39.5 | 14.2 | 3.3 | -20.1 | 9.1 |
| Las Yurdinas II | C/RS | LYII47 | 15108 | J (15±3) | F | Mandible | 9.1 | 36.7 | 12.7 | 3.4 | -20.2 | 9.0 |
| Las Yurdinas II | C/RS | LYII16 | 15081 | J (15±3) | F? | Mandible | 1.8 | *29.6* | *10.4* | 3.3 | -20.6 | 8.4 |
| Las Yurdinas II | C/RS | LYII17 | 15068 | J (15±3) | F? | Mandible | 3.5 | *44.0* | 16.0 | 3.2 | -20.0 | 9.4 |
| Las Yurdinas II | C/RS | LYII18 | 15075 | J (15±3) | F? | Mandible | 2.3 | 42.0 | 15.2 | 3.2 | -20.1 | 9.3 |
| Las Yurdinas II | C/RS | LYII28 | 15079 | J (15±3) | F? | Mandible | 3.3 | 39.2 | 14.2 | 3.2 | -19.9 | 9.3 |
| Las Yurdinas II | C/RS | LYII20 | 15073 | J (15±3) | ? | Mandible | 1.3 | *23.8* | *8.2* | 3.4 | -20.2 | 9.5 |
| Las Yurdinas II | C/RS | LYII21 | 15145 | J (15±3) | ? | Mandible | 2.2 | 41.3 | 14.9 | 3.2 | -20.4 | 8.2 |
| Las Yurdinas II | C/RS | LYII15 | 15087 | J (ca. 18) | M? | Mandible | 1.6 | 38.9 | 14.0 | 3.2 | -20.4 | 8.5 |
| Las Yurdinas II | C/RS | LYII34 | 15120 | YA | M | Mandible | 1.4 | 35.5 | 12.6 | 3.3 | -20.1 | 8.8 |
| Las Yurdinas II | C/RS | LYII29 | 15077 | YA | M | Mandible | 1.6 | 39.0 | 14.0 | 3.3 | -20.2 | 8.5 |
| Las Yurdinas II | C/RS | LYII26 | 15065 | YA | M | Mandible | 2.6 | 38.8 | 14.0 | 3.2 | -19.9 | 9.6 |
| Las Yurdinas II | C/RS | LYII25 | 15086 | YA | M | Mandible | 2.5 | 41.5 | 14.7 | 3.3 | -20.0 | 9.7 |
| Las Yurdinas II | C/RS | LYII27 | 15080 | YA | M | Mandible | 2.6 | *47.6* | *17.2* | 3.2 | -19.9 | 9.5 |
| Las Yurdinas II | C/RS | LYII30 | 15064 | YA | M | Mandible | 3.6 | 42.9 | 15.6 | 3.2 | -20.1 | 9.7 |
| Las Yurdinas II | C/RS | LYII40 | 15098 | YA | M? | Mandible | 7.2 | *45.0* | 15.9 | 3.3 | -20.2 | 8.3 |
| Las Yurdinas II | C/RS | LYII39 | 15094 | YA | F | Mandible | *0.4* | *11.0* | *3.2* | *4.0* | *-21.1* | *8.9* |
| Las Yurdinas II | C/RS | LYII41 | 15067 | YA | F | Mandible | 6.3 | 34.9 | 12.3 | 3.3 | -20.3 | 9.3 |
| Las Yurdinas II | C/RS | LYII56 | 15085 | YA | F | Mandible | 20.4 | *44.8* | *16.1* | 3.2 | -20.1 | 9.4 |
| Las Yurdinas II | C/RS | LYII43 | 15089 | YA | F | Mandible | 11.1 | 41.0 | 14.7 | 3.3 | -20.7 | 9.1 |
| Las Yurdinas II | C/RS | LYII38 | 15082 | YA | F | Mandible | 1.8 | 39.7 | 14.0 | 3.3 | -20.4 | 9.3 |
| Las Yurdinas II | C/RS | LYII42 | 15071 | YA | F | Mandible | 10.8 | 42.1 | 14.9 | 3.3 | -20.8 | 9.0 |
| Las Yurdinas II | C/RS | LYII44 | 15103 | YA | F | Mandible | 14.5 | 38.3 | 13.8 | 3.2 | -20.4 | 9.3 |
| Las Yurdinas II | C/RS | LYII65 | 15149 | YA | F? | Mandible | 12.7 | 40.8 | 14.5 | 3.3 | -19.4 | 10.4 |
| Las Yurdinas II | C/RS | LYII19 | 15078 | YA | F? | Mandible | 2.1 | 36.9 | 13.1 | 3.3 | -19.8 | 9.4 |
| Las Yurdinas II | C/RS | LYII35 | n/a | MA | M | Mandible | 1.7 | *29.7* | *10.5* | 3.3 | -20.3 | 9.1 |
| Las Yurdinas II | C/RS | LYII36 | 15102 | MA | M | Mandible | 1.3 | *28.3* | *9.7* | 3.4 | -19.8 | 9.3 |
| Las Yurdinas II | C/RS | LYII31 | 15113 | MA | M | Mandible | 1.4 | 39.9 | 13.8 | 3.4 | -20.5 | 9.6 |
| Las Yurdinas II | C/RS | LYII33 | 15074 | MA | M | Mandible | 2.5 | 40.6 | 14.6 | 3.3 | -20.4 | 9.5 |
| Las Yurdinas II | C/RS | LYII46 | 15115 | MA | F | Mandible | 5.7 | 34.4 | 12.0 | 3.3 | -20.0 | 9.1 |
| Las Yurdinas II | C/RS | LYII49 | 15066 | MA | F | Mandible | 9.8 | 38.7 | 13.8 | 3.3 | -20.6 | 8.9 |
| Las Yurdinas II | C/RS | LYII37 | 15069 | MA | F | Mandible | 3.5 | *45.7* | *16.5* | 3.2 | -20.2 | 9.4 |
| Las Yurdinas II | C/RS | LYII45 | 15095 | MA | F | Mandible | 5.8 | 38.8 | 13.7 | 3.3 | -19.9 | 8.6 |
| Las Yurdinas II | C/RS | LYII53 | n/a | MA | F | Mandible | 8.9 | 39.8 | 14.2 | 3.3 | -20.0 | 9.9 |
| Las Yurdinas II | C/RS | LYII24 | 15062 | OA | M | Mandible | 3.6 | 39.6 | 14.2 | 3.2 | -19.7 | 9.9 |
| Las Yurdinas II | C/RS | LYII32 | 15061 | OA | M | Mandible | 3.9 | 43.1 | 15.6 | 3.2 | -19.6 | 9.8 |
| Las Yurdinas II | C/RS | LYII50 | 15063 | OA | F | Mandible | 14.2 | 42.2 | 14.9 | 3.3 | -19.9 | 9.6 |
| Las Yurdinas II | C/RS | LYII51 | 15072 | OA | F | Mandible | 17.3 | 39.9 | 14.4 | 3.2 | -20.5 | 9.1 |
| Las Yurdinas II | C/RS | LYII48 | 15107 | IA | F | Mandible | 8.4 | 35.6 | 12.6 | 3.3 | -20.3 | 9.2 |
|  |  |  |  |  |  | *x̅* (n=48) | 6.0 | 39.3 | 14.0 | 3.3 | -20.1 | 9.2 |
|  |  |  |  |  |  | σ | 5.5 | 5.3 | 2.0 | <0.1 | 0.3 | 0.5 |
|  |  |  |  |  |  | Min. | 1.1 | 23.8 | 8.2 | 3.2 | -20.8 | 8.2 |
|  |  |  |  |  |  | Max. | 21.8 | 54.7 | 20.1 | 3.4 | -19.4 | 10.4 |
|  |  |  |  |  |  |  |  |  |  |  |  |  |
| Los Husos I | C/RS | LHI65 | n/a | C (8±2) | ? | Mandible | 5.9 | *22.2* | *7.5* | 3.4 | -20.2 | 9.1 |
| Los Husos I | C/RS | LHI62 | 282 | J (ca. 15) | F | Mandible | 2.5 | *26.0* | *9.0* | 3.4 | -19.9 | 9.4 |
| Los Husos I | C/RS | LHI58 | 342.5 | YA | M | Mandible | 2.7 | *26.0* | *8.9* | 3.4 | -20.1 | 9.3 |
| Los Husos I | C/RS | LHI63 | 299 | YA | M | Mandible | 4.1 | *28.6* | *10.0* | 3.3 | -19.9 | 9.6 |
| Los Husos I | C/RS | LHI60 | 360/80 | YA | ? | Mandible | 36.3 | 33.9 | 12.1 | 3.3 | -20.6 | 9.7 |
| Los Husos I | C/RS | LHI64 | 367 | YA | ? | Mandible | 1.9 | *23.9* | *8.3* | 3.4 | -20.4 | 9.3 |
| Los Husos I | C/RS | LHI59 | 300 | YA | ? | Mandible | 4.7 | *27.4* | *9.5* | 3.4 | -20.1 | 8.3 |
| Los Husos I | C/RS | LHI57 | 378 | MA | F? | Mandible | 3.2 | 34.2 | 12.2 | 3.3 | -20.2 | 9.1 |
| Los Husos I | C/RS | LHI61 | 382 | IA | M? | Mandible | *0.2* | *1.4* | *0.1* | *11.7* | *-26.0* | *0.3* |
|  |  |  |  |  |  | *x̅* (n=8) | 7.7 | 27.8 | 9.7 | 3.4 | -20.2 | 9.2 |
|  |  |  |  |  |  | σ | 11.6 | 4.3 | 1.7 | 0.1 | 0.3 | 0.4 |
|  |  |  |  |  |  | Min. | 1.9 | 22.2 | 7.5 | 3.3 | -20.6 | 8.3 |
|  |  |  |  |  |  | Max. | 36.6 | 34.2 | 12.2 | 3.5 | -19.9 | 9.7 |
|  |  |  |  |  |  |  |  |  |  |  |  |  |
| Peña Larga | C/RS | CPL3 | 20947 | C (7±2) | ? | Mandible | *0.1* | *2.7* | *0.3* | *12.2* | *-24.7* | *3.8* |
| Peña Larga | C/RS | CPL4 | 32037 | C (7±2) | ? | Mandible | *0.6* | *18.3* | *6.1* | 3.5 | -20.4 | 8.8 |
| Peña Larga | C/RS | CPL2 | 4968 | J (15±3) | ? | Maxilla | 10.9 | 38.9 | 13.9 | 3.3 | -20.3 | 9.5 |
| Peña Larga | C/RS | CPL11 | 47905 | YA | M | Mandible | *0.1* | *4.2* | *0.4* | *13.8* | *-23.8* | *7.0* |
| Peña Larga | C/RS | CPL5 | 50365 | YA | F? | Mandible | 1.8 | 31.5 | 11.1 | 3.3 | -20.5 | 10.1 |
| Peña Larga | C/RS | CPL8' | 7162 | MA | F | Mandible | 1.2 | *29.2* | *10.4* | 3.3 | -20.5 | 9.4 |
| Peña Larga | C/RS | CPL9 | 34133 | OA | F? | Mandible | 1.4 | *20.3* | *7.0* | 3.4 | -20.3 | 9.3 |
| Peña Larga | C/RS | CPL6' | 1199 | IA | M | Mandible | *0.4* | *4.7* | *0.4* | *12.9* | *-24.0* | *5.4* |
| Peña Larga | C/RS | CPL10 | 26638 | IA | F? | Mandible | *0.1* | *2.5* | *0.3* | *11.5* | *-25.2* | *3.8* |
| Peña Larga | C/RS | CPL12 | 24967 | IA | F? | Mandible | *0.1* | *3.2* | *0.2* | *19.0* | *-24.4* | *2.3* |
| Peña Larga | C/RS | CPL13 | 36043 | IA | F? | Mandible | *0.1* | *3.4* | *0.4* | *9.0* | *-23.2* | *5.0* |
| Peña Larga | C/RS | CPL14' | 35918 | IA | ? | Mandible | 1.3 | 36.1 | 12.9 | 3.3 | -20.2 | 9.5 |
| Peña Larga | C/RS | CPL15 | 48575 | IA | ? | Mandible | *0.1* | *3.9* | *0.3* | *17.2* | *-24.6* | *3.8* |
|  |  |  |  |  |  | *x̅ (n=6)* | 2.9 | 29.0 | 10.2 | 3.3 | -20.4 | 9.4 |
|  |  |  |  |  |  | *σ* | 4.0 | 8.3 | 3.2 | 0.1 | 0.1 | 0.4 |
|  |  |  |  |  |  | *Min.* | 0.6 | 18.3 | 6.1 | 3.3 | -20.5 | 8.8 |
|  |  |  |  |  |  | *Max.* | 10.9 | 38.9 | 14.0 | 3.5 | -20.2 | 10.1 |
|  |  |  |  |  |  |  |  |  |  |  |  |  |
| El Sotillo | M | ES103 | n/a | MA | ? | Mandible | 3.2 | 34.7 | 11.8 | 3.4 | -20.0 | 10.0 |
| El Sotillo | M | ES104 | n/a | MA | ? | Mandible | 2.6 | 33.0 | 11.5 | 3.4 | -20.1 | 9.8 |
|  |  |  |  |  |  | *x̅* (n=2) | 2.9 | 33.9 | 11.6 | 3.4 | -20.0 | 9.9 |
|  |  |  |  |  |  | σ | 0.3 | 1.2 | 0.2 | <0.1 | <0.1 | 0.1 |
|  |  |  |  |  |  | Min. | 2.6 | 33.0 | 11.5 | 3.4 | -20.1 | 9.8 |
|  |  |  |  |  |  | Max. | 3.2 | 34.7 | 11.8 | 3.4 | -20.0 | 10.0 |
|  |  |  |  |  |  |  |  |  |  |  |  |  |
| Alto de la Huesera | M | LHUE26 | 2237.10 | C (8±2) | ? | Mandible | 10.2 | 42.1 | 15.1 | 3.2 | -19.7 | 8.6 |
| Alto de la Huesera | M | LHUE54 | 2166.2 | C (8±2) | ? | Mandible | 7.4 | 41.5 | 14.8 | 3.3 | -19.5 | 9.0 |
| Alto de la Huesera | M | LHUE6c | 2436.2 | C (8±2) | ? | Mandible | 1.6 | 31.3 | *10.9* | 3.4 | -20.0 | 7.6 |
| Alto de la Huesera | M | LHUE27 | 2285.22 | C (9±2) | ? | Mandible | 3.5 | *29.6* | *10.5* | 3.3 | -20.6 | 8.6 |
| Alto de la Huesera | M | LHUE28 | 2308.1 | C (9±2) | ? | Mandible | 2.0 | *28.8* | *10.0* | 3.4 | -19.8 | 9.5 |
| Alto de la Huesera | M | LHUE55 | 2165.13 | C (11±2) | ? | Mandible | 2.8 | 32.2 | 11.4 | 3.3 | -19.7 | 8.1 |
| Alto de la Huesera | M | LHUE29 | 2283.44 | C (12±3) | ? | Mandible | 9.3 | *44.5* | *16.1* | 3.2 | -20.0 | 9.1 |
| Alto de la Huesera | M | LHUE48 | 2114 | C (12±3) | ? | Mandible | 5.7 | 39.4 | 14.1 | 3.3 | -20.3 | 9.0 |
| Alto de la Huesera | M | LHUE30 | 2209.6 | J (15±3) | ? | Mandible | 6.7 | 34.5 | 12.1 | 3.3 | -20.4 | 9.2 |
| Alto de la Huesera | M | LHUE31 | 2189.1 | J (ca. 18) | M | Mandible | 4.7 | 37.0 | 13.3 | 3.2 | -19.8 | 9.1 |
| Alto de la Huesera | M | LHUE51 | 2096.9 | YA | M | Mandible | 6.2 | 36.6 | 13.0 | 3.3 | -19.8 | 8.8 |
| Alto de la Huesera | M | LHUE32 | 2113 | YA | M | Mandible | 1.9 | 30.8 | *10.9* | 3.3 | -20.1 | 9.7 |
| Alto de la Huesera | M | LHUE2i | 2075 | YA | M | Mandible | 1.8 | 36.5 | 13.0 | 3.3 | -19.3 | 8.3 |
| Alto de la Huesera | M | LHUE16c | 2372.15 | YA | M | Mandible | 5.8 | 36.0 | 12.6 | 3.3 | -20.0 | 8.8 |
| Alto de la Huesera | M | LHUE49 | 2110 | YA | M? | Mandible | 3.6 | 31.5 | 11.2 | 3.3 | -20.5 | 8.7 |
| Alto de la Huesera | M | LHUE4c | 2459 | YA | M? | Mandible | *0.7* | 31.9 | 11.0 | 3.4 | -20.1 | 8.1 |
| Alto de la Huesera | M | LHUE36 | 2157 | YA | F | Mandible | 4.4 | 32.2 | 11.5 | 3.3 | -20.0 | 9.8 |
| Alto de la Huesera | M | LHUE34 | 2111 | YA | F | Mandible | *0.9* | *16.4* | *5.6* | 3.4 | -20.0 | 8.7 |
| Alto de la Huesera | M | LHUE52 | 2170 | YA | F? | Mandible | 9.4 | 41.0 | 14.6 | 3.3 | -19.6 | 9.1 |
| Alto de la Huesera | M | LHUE50 | 2095.8 | YA | F? | Mandible | 7.2 | 33.6 | 12.1 | 3.2 | -19.9 | 8.9 |
| Alto de la Huesera | M | LHUE13c | 2346.2 | YA | F? | Mandible | 1.5 | *29.2* | *9.9* | 3.4 | -20.1 | 8.7 |
| Alto de la Huesera | M | LHUE35 | 2112 | YA | ? | Mandible | 2.8 | *24.4* | *8.4* | 3.4 | -20.0 | 8.7 |
| Alto de la Huesera | M | LHUE37 | 2129.5 | YA | ? | Mandible | 7.4 | 37.4 | 13.2 | 3.3 | -19.2 | 9.7 |
| Alto de la Huesera | M | LHUE45 | 2334.46 | YA | ? | Mandible | 8.0 | 34.4 | 12.2 | 3.3 | -19.8 | 9.7 |
| Alto de la Huesera | M | LHUE5c | 2422.10 | YA | ? | Mandible | *0.7* | 33.2 | 11.3 | 3.4 | -20.2 | 9.3 |
| Alto de la Huesera | M | LHUE33 | 2189.7 | MA | M | Mandible | 1.9 | 39.5 | 13.9 | 3.3 | -20.0 | 10.1 |
| Alto de la Huesera | M | LHUE17c | 2382.2 | MA | M | Mandible | 4.6 | *28.8* | *9.9* | 3.4 | -19.7 | 8.8 |
| Alto de la Huesera | M | LHUE19c | 2386.2 | MA | M | Mandible | 7.1 | 37.9 | 13.3 | 3.3 | -19.6 | 9.5 |
| Alto de la Huesera | M | LHUE53 | 2169 | MA | M | Mandible | 8.7 | 36.9 | 13.2 | 3.3 | -19.5 | 9.8 |
| Alto de la Huesera | M | LHUE7c | 2435.2 | MA | M | Mandible | 3.6 | 37.9 | 13.5 | 3.3 | -20.2 | 7.8 |
| Alto de la Huesera | M | LHUE18c | 2384.3 | MA | M | Mandible | 6.2 | 35.9 | 12.6 | 3.3 | -19.7 | 9.1 |
| Alto de la Huesera | M | LHUE12c | 2335.2 | MA | M? | Mandible | 4.8 | 34.6 | 12.2 | 3.3 | -19.9 | 9.1 |
| Alto de la Huesera | M | LHUE62 | 2132.2 | MA | F | Mandible | 2.9 | *28.8* | *9.6* | 3.5 | -20.0 | 9.3 |
| Alto de la Huesera | M | LHUE15c | 2359.2 | MA | F | Mandible | 3.1 | *26.5* | *8.8* | 3.5 | -20.0 | 8.4 |
| Alto de la Huesera | M | LHUE14c | 2349.2 | MA | F? | Mandible | 4.5 | 34.8 | 12.0 | 3.4 | -19.6 | 8.9 |
| Alto de la Huesera | M | LHUE46 | 2141.17 | MA | ? | Mandible | 2.2 | *21.2* | *7.3* | 3.4 | -19.5 | 9.5 |
| Alto de la Huesera | M | LHUE40 | 2322.41 | MA | ? | Mandible | 3.4 | *25.9* | *8.9* | 3.4 | -19.5 | 10.1 |
| Alto de la Huesera | M | LHUE38 | 2133.6 | OA | M | Mandible | 1.1 | 41.8 | 14.6 | 3.3 | -20.3 | 9.4 |
| Alto de la Huesera | M | LHUE39 | 2107.9 | OA | M | Mandible | 2.5 | *24.2* | *8.5* | 3.3 | -20.4 | 9.1 |
| Alto de la Huesera | M | LHUE44 | 2213.1 | OA | ? | Mandible | 4.4 | 33.1 | 11.7 | 3.3 | -20.2 | 8.4 |
| Alto de la Huesera | M | LHUE41 | 2211 | IA | M? | Mandible | 1.3 | *12.7* | *4.3* | 3.5 | -19.9 | 9.6 |
| Alto de la Huesera | M | LHUE42 | 2305.25 | IA | F | Mandible | 3.6 | 41.3 | 14.8 | 3.3 | -19.6 | 9.7 |
| Alto de la Huesera | M | LHUE43 | 2311.2 | IA | F | Mandible | 1.6 | *16.3* | *5.6* | 3.4 | -19.8 | 10.2 |
| Alto de la Huesera | M | LHUE56 | 2168.29 | IA | F? | Mandible | 1.0 | *14.0* | *4.5* | 3.6 | -19.8 | 8.2 |
| Alto de la Huesera | M | LHUE3c' | 2432.5 | IA | F? | Mandible | 10.6 | 32.6 | 11.4 | 3.3 | -20.3 | 7.9 |
| Alto de la Huesera | M | LHUE47 | 2116.23 | IA | ? | Mandible | 1.0 | *21.6* | *7.2* | 3.5 | -19.5 | 8.8 |
|  |  |  |  |  |  | *x̅* (n=46) | 4.3 | 32.0 | 11.2 | 3.3 | -19.9 | 9.0 |
|  |  |  |  |  |  | σ | 2.8 | 7.6 | 2.8 | 0.1 | 0.3 | 0.6 |
|  |  |  |  |  |  | Min. | 0.7 | 12.7 | 4.3 | 3.2 | -20.6 | 7.6 |
|  |  |  |  |  |  | Max. | 10.6 | 44.5 | 16.2 | 3.6 | -19.2 | 10.2 |
|  |  |  |  |  |  |  |  |  |  |  |  |  |
| Chabola de la Hechicera | M | CH97 | 19.14 | C (8±2) | ? | Mandible | 10.2 | 40.6 | 14.4 | 3.3 | -20.1 | 8.7 |
| Chabola de la Hechicera | M | CH98 | 19.5 | C (10±2) | ? | Mandible | 11.4 | 41.3 | 14.9 | 3.2 | -19.6 | 8.8 |
| Chabola de la Hechicera | M | CH96 | 19.13 | YA | M | Mandible | 10.7 | 41.8 | 15.1 | 3.2 | -20.3 | 9.8 |
| Chabola de la Hechicera | M | CH93 | 19.18 | YA | M | Mandible | 11.8 | 42.2 | 14.4 | 3.4 | -20.8 | 9.6 |
| Chabola de la Hechicera | M | CH94 | 19.11.13 | YA | F? | Mandible | 5.8 | 35.0 | 12.5 | 3.3 | -20.3 | 9.3 |
| Chabola de la Hechicera | M | CH95 | 17.16.1 | YA | F? | Mandible | 7.6 | 40.3 | 14.2 | 3.3 | -20.4 | 8.1 |
|  |  |  |  |  |  | *x̅* (n=6) | 9.6 | 40.2 | 14.2 | 3.3 | -20.3 | 9.1 |
|  |  |  |  |  |  | σ | 2.4 | 2.6 | 0.9 | 0.1 | 0.4 | 0.6 |
|  |  |  |  |  |  | Min. | 5.8 | 35.0 | 12.5 | 3.2 | -20.8 | 8.1 |
|  |  |  |  |  |  | Max. | 11.8 | 42.2 | 15.1 | 3.4 | -19.6 | 9.8 |
|  |  |  |  |  |  |  |  |  |  |  |  |  |
| Longar | M | LON35 | 326 | C (7±2) | ? | Mandible | 1.6 | *24.4* | *8.4* | 3.4 | -20.4 | 9.0 |
| Longar | M | LON4 | 307 | C (8±2) | ? | Mandible | 4.9 | 35.8 | 12.7 | 3.3 | -19.6 | 9.2 |
| Longar | M | LON11 | 1862 | C (8±2) | ? | Mandible | 3.4 | 30.0 | *10.5* | 3.3 | -19.8 | 9.4 |
| Longar | M | LON2 | 2014 | C (9±2) | ? | Mandible | 3.8 | 34.5 | 12.1 | 3.3 | -20.2 | 9.1 |
| Longar | M | LON16 | 541 | C (9±2) | ? | Mandible | 2.0 | 36.9 | 12.7 | 3.4 | -20.1 | 9.1 |
| Longar | M | LON18 | 1980B | C (9±2) | ? | Mandible | 7.3 | 35.3 | 12.5 | 3.3 | -19.8 | 9.3 |
| Longar | M | LON26 | 1661 | C (9±2) | ? | Mandible | 1.2 | *24.3* | *8.2* | 3.5 | -20.3 | 8.7 |
| Longar | M | LON17 | 1980A | C (10±2) | ? | Mandible | 1.5 | *27.9* | *9.7* | 3.4 | -19.9 | 9.3 |
| Longar | M | LON21 | 930 | C (10±2) | ? | Mandible | 2.9 | 33.7 | 11.7 | 3.4 | -20.3 | 9.5 |
| Longar | M | LON6 | 430 | C (11±2) | ? | Mandible | 2.8 | *29.7* | *10.2* | 3.4 | -20.3 | 9.5 |
| Longar | M | LON22 | 1149 | C (11±2) | ? | Mandible | 5.5 | 32.0 | 11.3 | 3.3 | -19.9 | 9.4 |
| Longar | M | LON8 | 1600 | C (12±3) | ? | Mandible | 2.2 | *26.8* | *9.0* | 3.5 | -19.7 | 9.9 |
| Longar | M | LON28 | 1805 | C (12±3) | ? | Mandible | 3.6 | 34.3 | 12.1 | 3.3 | -19.7 | 10.2 |
| Longar | M | LON12 | 198 | YA | M | Mandible | 2.0 | *25.0* | *8.6* | 3.4 | -20.2 | 9.7 |
| Longar | M | LON34 | 209 | YA | M | Mandible | 1.7 | 31.2 | *10.5* | 3.5 | -19.8 | 9.5 |
| Longar | M | LON3 | 2060 | YA | M? | Mandible | 2.2 | *27.3* | *9.3* | 3.4 | -20.0 | 9.2 |
| Longar | M | LON36 | 866 | YA | M? | Mandible | 1.3 | *26.7* | *9.0* | 3.5 | -19.9 | 8.8 |
| Longar | M | LON9 | 1757A | YA | M? | Mandible | *0.9* | *18.8* | *6.1* | 3.6 | -20.3 | 8.9 |
| Longar | M | LON38 | 1967 | YA | M? | Mandible | 1.2 | *24.1* | *8.0* | 3.5 | -19.7 | 10.2 |
| Longar | M | LON42 | 1608 | YA | F | Mandible | *0.2* | *22.9* | *7.5* | *3.6* | *-20.2* | *9.2* |
| Longar | M | LON37 | 1351 | YA | F? | Mandible | 5.6 | 37.6 | 13.4 | 3.3 | -19.5 | 10.6 |
| Longar | M | LON19 | 640 | YA | F? | Mandible | 5.3 | 32.9 | 11.5 | 3.3 | -20.5 | 9.4 |
| Longar | M | LON30 | 1981 | YA | F? | Mandible | 1.2 | *20.6* | *6.9* | 3.5 | -20.2 | 9.1 |
| Longar | M | LON29 | 1847 | YA | F? | Mandible | 1.0 | *21.0* | *6.9* | 3.5 | -20.3 | 9.9 |
| Longar | M | LON33 | 194 | YA | F? | Mandible | 1.5 | *29.4* | *9.7* | 3.5 | -20.3 | 9.4 |
| Longar | M | LON41 | 919 | YA | F? | Mandible | 1.1 | *23.7* | *8.1* | 3.4 | -20.2 | 9.9 |
| Longar | M | LON39 | 1984 | YA | F? | Mandible | *0.9* | *20.8* | *6.8* | 3.6 | -20.3 | 9.5 |
| Longar | M | LON31 | 2008 | YA | ? | Mandible | 1.8 | *26.4* | *9.1* | 3.4 | -19.8 | 9.9 |
| Longar | M | LON7 | 1075 | YA | ? | Mandible | 3.2 | 33.8 | 12.0 | 3.3 | -19.8 | 9.5 |
| Longar | M | LON27 | 1687 | YA | ? | Mandible | 2.2 | *27.4* | *9.5* | 3.4 | -20.0 | 9.1 |
| Longar | M | LON20 | 105 | YA | ? | Mandible | 2.0 | *27.2* | *9.3* | 3.4 | -19.9 | 9.7 |
| Longar | M | LON15 | 1865 | MA | M | Mandible | *0.9* | *14.3* | *4.3* | *3.9* | *-20.2* | *9.0* |
| Longar | M | LON5 | 310 | MA | M | Mandible | 2.3 | 32.5 | 11.5 | 3.3 | -19.9 | 9.9 |
| Longar | M | LON43 | 730 | MA | M | Mandible | 2.3 | *29.0* | *10.1* | 3.3 | -19.8 | 10.1 |
| Longar | M | LON23 | 1356 | MA | M? | Mandible | 5.2 | 31.0 | *10.9* | 3.3 | -19.8 | 10.3 |
| Longar | M | LON32 | 104 | MA | F? | Mandible | 2.2 | 30.7 | *10.6* | 3.4 | -19.9 | 9.5 |
| Longar | M | LON40 | 1560 | MA | F? | Mandible | 1.8 | *28.4* | *9.8* | 3.4 | -19.5 | 9.7 |
| Longar | M | LON14 | 1801 | MA | ? | Mandible | 1.1 | *18.8* | *6.0* | 3.6 | -20.0 | 10.2 |
| Longar | M | LON10 | 1757B | IA | F | Mandible | 2.6 | *27.9* | *9.5* | 3.4 | -20.3 | 9.7 |
| Longar | M | LON24 | 1524 | IA | F? | Mandible | 1.6 | *22.9* | *7.7* | 3.5 | -20.4 | 9.1 |
| Longar | M | LON25 | 1535 | IA | F? | Mandible | 2.7 | *29.5* | *10.2* | 3.4 | -19.9 | 9.8 |
|  |  |  |  |  |  | *x̅* (n=39) | 2.5 | 28.5 | 9.8 | 3.4 | -20.0 | 9.5 |
|  |  |  |  |  |  | σ | 1.5 | 5.0 | 1.9 | 0.1 | 0.3 | 0.4 |
|  |  |  |  |  |  | Min. | 0.9 | 18.8 | 6.0 | 3.3 | -20.5 | 8.7 |
|  |  |  |  |  |  | Max. | 7.3 | 37.6 | 13.4 | 3.6 | -19.5 | 10.6 |

**^1^***C/RS* = cave/rockshelter; *M* = megalithic grave.

^2^ Inventory number. *n/a* = not available.

**^3^***C* = child; *J* = juvenile; *YA* = young adult; *MA* = mature adult; *OA* = older adult; *IA* = indeterminate adult.

**^4^***M* = male; *M?* = probable male; *F* = female; *F?* = probable female; *?* = ambiguous/indeterminate.

^5^ Values from samples exhibiting anomalous C:N ratios are shown in gray italics. Anomalous indicators from other samples are shown in italics.
