# Supplementary material for: Living different lives: Early social differentiation identified through linking mortuary and isotopic variability in Late Neolithic/ Early Chalcolithic north-central Spain
Source: PLoS One. 2017 Sep 27;12(9):e0177881. doi: 10.1371/journal.pone.0177881 (PMC5643145; doi:10.1371/journal.pone.0177881)
Supplement: S2 Table — (DOCX) [file pone.0177881.s009.docx]

| **S2 Table. Faunal isotope values and bone collagen quality indicators of the samples analyzed.** | | | | | | | | | | | | |
| --- | --- | --- | --- | --- | --- | --- | --- | --- | --- | --- | --- | --- |
| Site | Type^1^ | Sample | Inv.^2^ | Species | Age^3^ | Element | %Col^4^ | %C^4^ | %N^4^ | C:N^4^ | δ^13^C (‰) | δ^15^N (‰) |
| Los Husos I | C/RS | LHI67 | 353/59 | *Bos taurus* | ? | Humerus | 16.3 | *20.1* | *6.9* | 3.4 | -20.6 | 4.1 |
| Los Husos I | C/RS | LHI70 | 218 | *Bos taurus* | ? | Mandible | 2.9 | *23.3* | *8.1* | 3.4 | -21.1 | 4.1 |
| Los Husos I | C/RS | LHI73 | 232 | *Bos taurus* | NA | Tibia | 17.1 | 40.2 | 14.4 | 3.3 | -20.7 | 5.7 |
| Los Husos I | C/RS | LHI71 | 401 | *Ovis aries/Capra hircus* | ? | Femur | 9.0 | 39.2 | 13.9 | 3.3 | -20.5 | 3.9 |
| Los Husos I | C/RS | LHI72 | 372 | *Ovis aries/Capra hircus* | ? | Femur | 18.2 | 41.7 | 15.0 | 3.3 | -20.4 | 6.6 |
| Los Husos I | C/RS | LHI76 | 226 | *Ovis aries/Capra hircus* | NA | Mandible | 5.1 | 41.8 | 14.9 | 3.3 | -21.1 | 3.3 |
| Los Husos I | C/RS | LHI77 | 274 | *Ovis aries/Capra hircus* | NA | Mandible | 4.1 | 42.1 | 14.8 | 3.3 | -20.7 | 5.5 |
| Los Husos I | C/RS | LHI78 | 229 | *Ovis aries/Capra hircus* | NA | Mandible | 3.7 | 37.6 | 13.4 | 3.3 | -20.9 | 6.2 |
| Los Husos I | C/RS | LHI79 | 225 | *Ovis aries/Capra hircus* | ? | Mandible | *1.4* | *19.1* | *6.1* | *3.7* | *-21.0* | *4.5* |
| Los Husos I | C/RS | LHI80 | 200A | *Ovis aries/Capra hircus* | NA | Mandible | 2.0 | 39.0 | 13.4 | 3.4 | -20.9 | 4.4 |
| Los Husos I | C/RS | LHI81 | 200B | *Ovis aries/Capra hircus* | NA | Mandible | 7.4 | 39.3 | 14.0 | 3.3 | -20.4 | 5.1 |
| Los Husos I | C/RS | LHI82 | 201 | *Ovis aries/Capra hircus* | NA | Mandible | *0.8* | 37.3 | 12.9 | 3.4 | -20.9 | 4.7 |
| Los Husos I | C/RS | LHI66 | 371 | *Cervus elaphus* | A | Femur | 53.6 | 40.7 | 14.5 | 3.3 | -19.8 | 6.4 |
| Los Husos I | C/RS | LHI74 | 210A | *Cervus elaphus* | ? | Mandible | 5.7 | *18.2* | *6.0* | 3.5 | -21.3 | 3.7 |
| Los Husos I | C/RS | LHI75 | 210B | *Cervus elaphus* | NA | Mandible | *0.7* | *19.8* | *6.6* | 3.5 | -20.6 | 7.2 |
| Los Husos I | C/RS | LHI83 | 254 | *Sus domesticus* | NA | Mandible | 11.1 | 35.6 | 12.0 | 3.5 | -20.8 | 5.0 |
| Los Husos I | C/RS | LHI84 | 290 | *Sus domesticus* | ? | Mandible | 16.3 | 42.8 | 15.2 | 3.3 | -21.1 | 8.7 |
| Los Husos I | C/RS | LHI85 | 200C | *Sus domesticus* | ? | Mandible | 3.3 | *28.5* | *9.9* | 3.4 | -20.3 | 5.6 |
| Los Husos I | C/RS | LHI86 | 208 | *Sus domesticus* | ? | Mandible | 1.9 | 33.3 | 11.3 | 3.4 | -19.7 | 2.6 |
|  |  |  |  |  |  |  |  |  |  |  |  |  |
| Peña Larga | C/RS | CPL16 | 50222 | *Bos taurus* | A | Mandible | 4.5 | 37.7 | 13.3 | 3.3 | -21.4 | 3.3 |
| Peña Larga | C/RS | CPL17 | 48446 | *Bos taurus* | A | Mandible | *0.1* | *3.7* | *0.4* | *12.4* | *-25.2* | *5.9* |
| Peña Larga | C/RS | CPL18 | 42784 | *Ovis aries/Capra hircus* | NA | Humerus | *0.8* | *27.5* | *9.4* | 3.4 | -20.3 | 3.8 |
| Peña Larga | C/RS | CPL24 | 42956 | *Cervus elaphus* | A | Carpus | 17.2 | 42.5 | 15.1 | 3.3 | -20.5 | 3.6 |
| Peña Larga | C/RS | CPL22 | 47597 | *Sus domesticus* | ? | Cranium | *8.9* | *3.1* | *0.2* | *17.8* | *-24.7* | *3.0* |
| Peña Larga | C/RS | CPL23 | 50026 | *Sus scrofa* | NA | Radius | 16.6 | 41.1 | 14.8 | 3.3 | -20.7 | 6.7 |
|  |  |  |  |  |  |  |  |  |  |  |  |  |
| El Sotillo | M | ES49 | 23237 | *Bos taurus* | ? | Humerus | 1.0 | 41.8 | 13.7 | 3.5 | -20.9 | 6.1 |
|  |  |  |  |  |  |  |  |  |  |  |  |  |
| Alto de la Huesera | M | LHUE57 | 2249 | *Bos taurus* | A | Femur | 7.8 | 41.3 | 14.9 | 3.2 | -20.5 | 4.3 |
| Alto de la Huesera | M | LHUE64 | 2270 | *Ovis aries/Capra hircus* | A | Metapodial | 6.5 | 43.2 | 15.4 | 3.3 | -20.2 | 4.9 |
|  |  |  |  |  |  |  |  |  |  |  |  |  |
| Chabola de la Hechicera | M | CH99 | n/a | *Bos taurus* | ? | Rib | 4.9 | 41.0 | 14.9 | 3.2 | -20.2 | 5.9 |
| Chabola de la Hechicera | M | CH100 | 23123A | *Ovis aries/Capra hircus* | ? | Humerus | 8.6 | 43.7 | 15.9 | 3.2 | -20.5 | 7.8 |
| Chabola de la Hechicera | M | CH101 | 23123B | *Ovis aries/Capra hircus* | ? | Humerus | 11.8 | 43.0 | 15.7 | 3.2 | -20.6 | 4.8 |
| Chabola de la Hechicera | M | CH102 | 23123C | *Sus domesticus* | ? | Mandible | 4.5 | 33.5 | 11.8 | 3.3 | -20.6 | 6.1 |

**^1^***C/RS* = cave/rockshelter; *M* = megalithic grave.

^2^ Inventory number. *n/a* = not available.

**^3^***NA* = non-adult; *A* = adult; *?* = indeterminate.

^4^ Values from samples exhibiting anomalous C:N ratios are shown in gray italics. Anomalous indicators from other samples are shown in italics.
