# Supplementary material for: Living different lives: Early social differentiation identified through linking mortuary and isotopic variability in Late Neolithic/ Early Chalcolithic north-central Spain
Source: PLoS One. 2017 Sep 27;12(9):e0177881. doi: 10.1371/journal.pone.0177881 (PMC5643145; doi:10.1371/journal.pone.0177881)
Supplement: S3 Table — (DOCX) [file pone.0177881.s010.docx]

| **S3 Table. Post hoc Tukey HSD tests used to assess heterogeneity in δ^13^C and δ^15^N values between sites.** | | | | | | | |
| --- | --- | --- | --- | --- | --- | --- | --- |
| δ^13^C | | | | | | | |
| Site (type^1^) | Las Yurdinas II (C/RS) | Los Husos I (C/RS) | Peña Larga (C/RS) | El Sotillo (M) | Alto de la Huesera (M) | Chabola de la Hechicera (M) | Longar (M) |
| Las Yurdinas II (C/RS) | - | 1.000 | 0.589 | 0.999 | **0.002** | 0.948 | 0.318 |
| Los Husos I (C/RS) | 1.000 | - | 0.898 | 0.997 | 0.199 | 0.996 | 0.745 |
| Peña Larga (C/RS) | 0.589 | 0.898 | - | 0.832 | **0.008** | 0.998 | 0.085 |
| El Sotillo (M) | 0.999 | 0.997 | 0.832 | - | 0.995 | 0.962 | 1.000 |
| Alto de la Huesera (M) | **0.002** | 0.199 | **0.008** | 0.995 | - | 0.065 | 0.696 |
| Chabola de la Hechicera (M) | 0.948 | 0.996 | 0.998 | 0.962 | 0.065 | - | 0.371 |
| Longar (M) | 0.318 | 0.745 | 0.085 | 1.000 | 0.696 | 0.371 | - |
| δ^15^N | | | | | | | |
| Site | Las Yurdinas II (C/RS) | Los Husos I (C/RS) | Peña Larga (C/RS) | El Sotillo (M) | Alto de la Huesera (M) | Chabola de la Hechicera (M) | Longar (M) |
| Las Yurdinas II (C/RS) | - | 1.000 | 0.916 | 0.529 | 0.587 | 0.996 | **0.028** |
| Los Husos I (C/RS) | 1.000 | - | 0.990 | 0.713 | 0.897 | 0.994 | 0.727 |
| Peña Larga (C/RS) | 0.916 | 0.990 | - | 0.949 | 0.443 | 0.844 | 0.999 |
| El Sotillo (M) | 0.529 | 0.713 | 0.949 | - | 0.237 | 0.453 | 0.976 |
| Alto de la Huesera (M) | 0.587 | 0.897 | 0.443 | 0.237 | - | 1.000 | **0.000** |
| Chabola de la Hechicera (M) | 0.996 | 0.994 | 0.844 | 0.453 | 1.000 | - | 0.311 |
| Longar (M) | **0.028** | 0.727 | 0.999 | 0.976 | **0.000** | 0.311 | - |

**^1^***C/RS* = cave/rockshelter; *M* = megalithic grave.
