# Supplementary material for: Living different lives: Early social differentiation identified through linking mortuary and isotopic variability in Late Neolithic/ Early Chalcolithic north-central Spain
Source: PLoS One. 2017 Sep 27;12(9):e0177881. doi: 10.1371/journal.pone.0177881 (PMC5643145; doi:10.1371/journal.pone.0177881)
Supplement: S4 Table — (DOCX) [file pone.0177881.s011.docx]

| **S4 Table. Summary statistics of human isotopic values grouped in non-adult (7-20 years) and adult (>20 years) age categories and statistical results obtained from comparing the mean values of both groups among sites, by site-type and in total.** | | | | | | | | | | | | | | | | | | | | | | | | | | |
| --- | --- | --- | --- | --- | --- | --- | --- | --- | --- | --- | --- | --- | --- | --- | --- | --- | --- | --- | --- | --- | --- | --- | --- | --- | --- | --- |
| Site (type^1^) | | δ^13^C | | | | | | | | | | | | | | δ^15^N | | | | | | | | | | |
|  |  | Non-adults | | | | | | Adults | | | | | | T test | | Non-adults | | | | Adults | | | | | T test | |
|  |  | *x̅* | | σ | | n | | *x̅* | | σ | | n | |  |  | *x̅* | | σ | n | *x̅* | | σ | | n |  |  |
| Las Yurdinas II (C/RS) | | -20.1 | | 0.2 | | 19 | | -20.1 | | 0.3 | | 29 | | *t* = 0.133  df = 46  *p* = 0.894 | | 9.0 | | 0.4 | 19 | 9.3 | | 0.5 | | 29 | *t* = 2.695  df = 46  ***p* = 0.010** | |
| Los Husos I (C/RS) | | -20.0 | | 0.2 | | 2 | | -20.2 | | 0.3 | | 6 | | *t* = 0.755  df = 6  *p* = 0.479 | | 9.3 | | 0.2 | 2 | 9.2 | | 0.5 | | 6 | *t* = 0.128  df = 6  *p* = 0.902 | |
| Peña Larga (C/RS) | | -20.4 | | 0.1 | | 2 | | -20.4 | | 0.2 | | 4 | | *t* = 0.037  df = 4  *p* = 0.972 | | 9.2 | | 0.4 | 2 | 9.6 | | 0.3 | | 4 | *t* = 1.346  df = 4  *p* = 0.250 | |
| El Sotillo (M) | | - | | - | | - | | -20.0 | | <0.1 | | 2 | | - | | - | | - | - | 9.9 | | 0.1 | | 2 | - | |
| Alto de la Huesera (M) | | -20.0 | | 0.3 | | 10 | | -19.9 | | 0.3 | | 36 | | *t* = 0.765  df = 44  *p* = 0.448 | | 8.8 | | 0.6 | 10 | 9.1 | | 0.6 | | 36 | *t* = 1.328  df = 44  *p* = 0.191 | |
| Chabola de la Hechicera (M) | | -19.9 | | 0.3 | | 2 | | -20.5 | | 0.3 | | 4 | | *t* = 2.330  df = 4  *p* = 0.080 | | 8.8 | | 0.1 | 2 | 9.2 | | 0.8 | | 4 | *t* = 0.765  df = 4  *p* = 0.487 | |
| Longar (M) | | -20.0 | | 0.3 | | 13 | | -20.0 | | 0.3 | | 26 | | *t* = 0.004  df = 37  *p* = 0.997 | | 9.3 | | 0.4 | 13 | 9.6 | | 0.4 | | 26 | *t* = 2.076  df = 37  ***p* = 0.045** | |
|  | | | | | | | | | | | | | | | | | | | | | | | | | | |
| Caves combined | -20.1 | | 0.2 | | 23 | | -20.2 | | 0.3 | | 39 | | *t* = 0.419  df = 60  *p* = 0.677 | | 9.0 | | 0.4 | | 23 | | 9.3 | | 0.4 | 39 | | *t* = 2.821  df = 60  ***p* = 0.006** |
| Monuments combined | -20.0 | | 0.3 | | 25 | | -20.0 | | 0.3 | | 68 | | *t* = 0.196  df = 91  *p* = 0.845 | | 9.1 | | 0.5 | | 25 | | 9.3 | | 0.6 | 68 | | *t* = 1.798  df = 91  *p* = 0.076 |
|  | | | | | | | | | | | | | | | | | | | | | | | | | | |
| Combined sample | -20.1 | | 0.3 | | 48 | | -20.0 | | 0.3 | | 107 | | *t* = 0.303  df = 153  *p* = 0.763 | | 9.0 | | 0.5 | | 48 | | 9.3 | | 0.6 | 107 | | *t* = 3.031  df = 153  ***p* = 0.003** |

**^1^***C/RS* = cave/rockshelter; *M* = megalithic grave.
