# Supplementary material for: Living different lives: Early social differentiation identified through linking mortuary and isotopic variability in Late Neolithic/ Early Chalcolithic north-central Spain
Source: PLoS One. 2017 Sep 27;12(9):e0177881. doi: 10.1371/journal.pone.0177881 (PMC5643145; doi:10.1371/journal.pone.0177881)
Supplement: S5 Table — (DOCX) [file pone.0177881.s012.docx]

| **S5 Table. Summary statistics of human isotopic values grouped in specific age categories (7-9, 10-12, 13-19, 20-39, ≥40).** | | | | | | | | | | | | | | | | | | |
| --- | --- | --- | --- | --- | --- | --- | --- | --- | --- | --- | --- | --- | --- | --- | --- | --- | --- | --- |
| Age group | δ^13^C | | | | | | | | | δ^15^N | | | | | | | | |
|  | Caves | | | Monuments | | | Total | | | Caves | | | Monuments | | | Total | | |
|  | *x̅* | σ | n | *x̅* | σ | n | *x̅* | σ | n | *x̅* | σ | n | *x̅* | σ | n | *x̅* | σ | n |
| 7-9 | -20.1 | 0.2 | 8 | -20.0 | 0.3 | 13 | -20.0 | 0.3 | 21 | 9.0 | 0.4 | 8 | 8.9 | 0.5 | 13 | 8.9 | 0.4 | 21 |
| 10-12 | -20.0 | 0.2 | 5 | -19.9 | 0.3 | 10 | -20.0 | 0.3 | 15 | 9.0 | 0.4 | 5 | 9.3 | 0.6 | 10 | 9.2 | 0.5 | 15 |
| 13-19 | -20.2 | 0.2 | 10 | -20.1 | 0.4 | 2 | -20.2 | 0.3 | 12 | 9.0 | 0.5 | 10 | 9.2 | 0.0 | 2 | 9.1 | 0.5 | 12 |
| 20-39 | -20.2 | 0.3 | 21 | -20.0 | 0.3 | 36 | -20.1 | 0.3 | 57 | 9.3 | 0.5 | 21 | 9.3 | 0.6 | 36 | 9.3 | 0.6 | 57 |
| ≥40 | -20.1 | 0.3 | 16 | -19.9 | 0.3 | 23 | -20.0 | 0.3 | 39 | 9.4 | 0.4 | 16 | 9.4 | 0.6 | 23 | 9.4 | 0.5 | 39 |
