# Supplementary material for: Living different lives: Early social differentiation identified through linking mortuary and isotopic variability in Late Neolithic/ Early Chalcolithic north-central Spain
Source: PLoS One. 2017 Sep 27;12(9):e0177881. doi: 10.1371/journal.pone.0177881 (PMC5643145; doi:10.1371/journal.pone.0177881)
Supplement: S6 Table — (DOCX) [file pone.0177881.s013.docx]

| **S6 Table. Statistical results obtained from comparing the mean values between non-adults (7-20 years) and between adults (>20 years) separately between sites and by site-type (see S4 table for the summary statistics of the groups being compared).** | | | | | |
| --- | --- | --- | --- | --- | --- |
| Comparison | Test | Non-adults | | Adults | |
|  |  | δ^13^C | δ^15^N | δ^13^C | δ^15^N |
| Between sites | One-way ANOVA | *F* _(5, 42)_ = 1.2  *p =* 0.314 | *F* _(5, 42)_ = 2.4  *p =* 0.054 | *F* _(6, 100)_ = 4.7  ***p* < 0.001** | *F* _(6, 100)_ = 3.6  ***p*** *=* **0.003** |
|  |  |  |  |  |  |
| Caves vs. Monuments | T-test | *t* = 2.117  df = 46  ***p* = 0.040** | *t* = 0.379  df = 46  *p* = 0.707 | *t* = 3.339  df = 105  ***p* = 0.001** | *t* = 0.124  df = 100.5  *p* = 0.902 |
