# Supplementary material for: Living different lives: Early social differentiation identified through linking mortuary and isotopic variability in Late Neolithic/ Early Chalcolithic north-central Spain
Source: PLoS One. 2017 Sep 27;12(9):e0177881. doi: 10.1371/journal.pone.0177881 (PMC5643145; doi:10.1371/journal.pone.0177881)
Supplement: S7 Table — (DOCX) [file pone.0177881.s014.docx]

| **S7 Table. Summary statistics of human isotopic values grouped according to sex estimation and statistical results obtained from comparing the mean values of males and females among sites, by site-type and in total.** | | | | | | | | | | | | | | | | | | | |
| --- | --- | --- | --- | --- | --- | --- | --- | --- | --- | --- | --- | --- | --- | --- | --- | --- | --- | --- | --- |
| Site (type^1^) | δ^13^C | | | | | | | | | | δ^15^N | | | | | | | | |
|  | Males | | | | Females | | | | T test | | Males | | | | | Females | | | T/U test |
|  | *x̅* | | σ | n | *x̅* | | σ | n |  |  | *x̅* | | σ | n | | *x̅* | σ | n |  |
| Las Yurdinas II (C/RS) | -20.1 | | 0.3 | 14 | -20.2 | | 0.3 | 21 | *t* = 0.865  df = 33  *p* = 0.393 | | 9.3 | | 0.5 | 14 | | 9.3 | 0.4 | 21 | *t* = 0.145  df = 33  *p* = 0.885 |
| Los Husos I (C/RS) | -20.0 | | 0.2 | 2 | -20.0 | | 0.2 | 2 | *t* = 0.188  df = 1.9  *p* = 0.869 | | 9.4 | | 0.2 | 2 | | 9.3 | 0.2 | 2 | *t* = 0.780  df = 2  *p* = 0.517 |
| Peña Larga (C/RS) | - | | - | - | -20.4 | | 0.1 | 3 | - | | - | | - | - | | 9.6 | 0.4 | 3 | - |
| El Sotillo (M) | - | | - | - | - | | - | - | - | | - | | - | - | | - | - | - | - |
| Alto de la Huesera (M) | -19.9 | | 0.3 | 17 | -19.9 | | 0.2 | 12 | *t* = 0.224  df = 27  *p* = 0.809 | | 9.0 | | 0.6 | 17 | | 9.0 | 0.7 | 12 | *t* = 0.256  df = 27  *p* = 0.800 |
| Chabola de la Hechicera (M) | -20.6 | | 0.4 | 2 | -20.4 | | <0.1 | 2 | *t* = 0.672  df = 1  *p* = 0.623 | | 9.7 | | 0.1 | 2 | | 8.7 | 0.9 | 2 | *t* = 1.664  df = 1  *p* = 0.338 |
| Longar (M) | -19.9 | | 0.2 | 9 | -20.1 | | 0.3 | 12 | *t* = 1.391  df = 19  *p* = 0.180 | | 9.6 | | 0.6 | 9 | | 9.6 | 0.4 | 12 | *t* = 0.107  df = 19  *p* = 0.916 |
|  | |  | | | |  | | | |  | |  | | |  | | | |  |
| Caves combined | -20.1 | | 0.3 | 16 | -20.2 | | 0.3 | 26 | *t* = 1.266  df = 40  *p* = 0.213 | | 9.3 | | 0.5 | 16 | | 9.3 | 0.4 | 26 | *U* = 174  *Z* = 0.881  *p* = 0.378 |
| Monuments combined | -20.0 | | 0.3 | 28 | -20.0 | | 0.3 | 26 | *t* = 0.657  df = 52  *p* = 0.514 | | 9.3 | | 0.6 | 28 | | 9.3 | 0.7 | 26 | *t* = 0.068  df = 52  *p* = 0.946 |
|  | | | | | | | | | | | | | | | | | | | |
| Combined sample | -20.0 | | 0.3 | 44 | -20.1 | | 0.3 | 52 | *t* = 1.605  df = 94  *p* = 0.112 | | 9.3 | | 0.6 | 44 | | 9.3 | 0.5 | 52 | *t* = 0.025  df = 94  *p* = 0.980 |

**^1^***C/RS* = cave/rockshelter; *M* = megalithic grave.
