# Supplementary material for: Living different lives: Early social differentiation identified through linking mortuary and isotopic variability in Late Neolithic/ Early Chalcolithic north-central Spain
Source: PLoS One. 2017 Sep 27;12(9):e0177881. doi: 10.1371/journal.pone.0177881 (PMC5643145; doi:10.1371/journal.pone.0177881)
Supplement: S8 Table — (DOCX) [file pone.0177881.s015.docx]

| **S8 Table. Statistical results obtained from comparing the mean values between males and between females separately between sites and by site-type (see S7 table for the summary statistics of the groups being compared).** | | | | | |
| --- | --- | --- | --- | --- | --- |
| Comparison | Test | Males | | Females | |
|  |  | δ^13^C | δ^15^N | δ^13^C | δ^15^N |
| Between sites | One-way ANOVA | *F* _(4, 39)_ = 2.7  ***p* = 0.047** | *F* _(4, 39)_ = 2.0  *p =* 0.118 | *F* _(5, 46)_ = 2.3  *p =* 0.063 | *F* _(5, 46)_ = 3.0  ***p* = 0.020** |
|  |  |  |  |  |  |
| Caves vs. monuments | T/U test | *t* = 1.112  df = 42  *p* = 0.272 | *t* = 0.136  df = 42  *p* = 0.892 | *t* = 1.973  df = 50  *p* = 0.054 | *U* = 324.5  *Z* = 0.247  *p* = 0.805 |
