# Supplementary material for: Living different lives: Early social differentiation identified through linking mortuary and isotopic variability in Late Neolithic/ Early Chalcolithic north-central Spain
Source: PLoS One. 2017 Sep 27;12(9):e0177881. doi: 10.1371/journal.pone.0177881 (PMC5643145; doi:10.1371/journal.pone.0177881)
Supplement: S9 Table — (DOCX) [file pone.0177881.s016.docx]

| **S9 Table. Minimum Number of Individuals (MNI) of the ungulate remains recovered from the sites analyzed.** | | | | | |
| --- | --- | --- | --- | --- | --- |
| Species | Caves | | Megalithic graves | | |
|  | Los Husos I  (layer III)^1^ | Peña Larga  (layer III)^2^ | El Sotillo^3^ | Alto de la Huesera  (lower layer/corridor)^3^ | Chabola de la Hechicera^3^ |
| *Bos taurus* | 5 | 2 | 1 | 2 | 1 |
| *Ovis aries / Capra hircus* | 9 | 3 | - | 1 | 2 |
| *Equus caballus* | 2 | - | - | - | - |
| *Cervus elaphus* | 3 | 1 | - | - | - |
| *Capreolus capreolus* | 2 | - | - | - | - |
| *Sus domesticus* | 4 | 3 | - | - | 1 |
| *Sus scropha* | 1 | 1 | - | - | - |

^1^ [43].

^2^ [44].

^3^ J. Fernández-Eraso and J. Mujika, pers. comm.
